# Semi-empirical supported, Ab initio derived thermodynamic properties for ClO_2_ and its sub and extended species, applied in water treatment cycles

**DOI:** 10.1016/j.heliyon.2024.e38796

**Published:** 2024-10-05

**Authors:** Natasha Misheer, Patrick G. Ndungu, Jan A. Pretorius

**Affiliations:** a The Department of Chemistry, University of Pretoria. Private Bag X20, Hatfield, Zip Code 0028, South Africa; b ESKOM Research and Innovation Centre, Rosherville, Private Bag 40157, Cleveland, Johannesburg, 2022, South Africa; c Centre for the Advancement of Scholarship, University of Pretoria, Private Bag X20, Hatfield, Zip Code 0028, South Africa

**Keywords:** NOM (natural organic matter), ClO_2_ (chlorine dioxide), GEMC (gibbs ensemble Monte Carlo), Semi-empirical quantum, Radicals, Ions

## Abstract

This paper describes a group of sixty (60) sub and extended chlorine oxide species with the general formulae of Cl_x_O_y_ (with x ≤ 2, y ≤ 8). Their role in water treatment cycles, behaving as key reactive species, is represented by a complex sequence of chemical inter-dependencies, exposed as a cohesive set of chemical reactions to demonstrate their cyclic role in aqueous media. An empirical/semi-empirical computational approach, supported by Ab Initio simulations, in accordance with open-shell character, has been followed to determine their optimum molecular geometries, to obtain their thermochemical properties. Besides a single molecular analysis, Grand Canonical Ensemble simulations, supported by a revised library of force field parameters, constituted a core component of the computational approach and proved to be invaluable in confirming thermochemical properties. This approach also offered finite estimates of optimum model sizes, a benefit with wider modelling application. Extended molecular species of ClO_2_ display a complex sequence of bonding character, with a variable charge dissipation (reported as partial charges), which complicates selection of basis sets in optimizing molecular geometries, during Ab Initio analyses.

Optimum molecular geometries were obtained using Gaussian and MOPAC, which in turn resulted in reliable Heats of Formation. These correlated well with energies extracted from the open literature. Thermodynamic Analysis of the reaction of selected chlorine oxides with water using FactSage, predicted the production of known and two previously undetected chlorine species, [ClO_4_]^-^ and [ClOH_2_]^+^.

## 1 Introduction

The removal of Natural Organic Matter (NOM) from water has become increasingly difficult due to the wide variety and complexity of organic compounds that make up NOM. These organic compounds within NOM can include humic substances (both soluble and insoluble fractions), aromatic compounds, proteins, lipids, waxes, small organic molecules, microbial debris, and various other organic compounds from the weathering or decay of living material [1,2]. Besides the complex composition of NOM, the change in seasons, temperature, pH, associated biological processes in a water body, and the water chemistry can affect the levels and constituent organic molecules that make-up NOM, which in turn affects water treatment cycles [2]. Coagulation and flocculation steps remain a key part of water treatment cycles globally; however, coagulation processes are typically efficient at the removal of hydrophobic and high molecular fractions of NOM [[3], [4], [5]]. Thus, any remaining components of NOM species remain in the treated water and can interfere with any subsequent treatment processes, especially chlorination or advanced oxidation steps, which results in toxic disinfection by-products [1,2,6,7].

Chlorination, using chlorine gas, remains a widely used key step for potable water and wastewater treatment cycles [6,[8], [9], [10]]. Chlorine gas is typically housed and dosed out of gas cylinders, and this does represent a safety risk in terms of accidental exposure, leaks, or explosions. Another key concern with the use of chlorine gas is its tendency to produce halogenated disinfection by-products, especially with residual NOM species in the water. An alternative to chlorine gas is the use of chlorine dioxide (ClO_2_), which has a few advantages over chlorine gas; such as, it typically produces minimal levels of toxic halogenated disinfection by-products, it's much more effective in inactivating viruses and bacteria, it is effective in the removal of algal contaminants, it reduces the levels of iron and manganese, it can be used over a wider range of pH, lower concentrations of ClO_2_ are needed when compared to Cl_2_, and it is usually generated on-site [6,8,[10], [11], [12], [13]].

However, despite its many advantages when compared to Cl_2_, ClO_2_ has some drawbacks including the formation of chlorite and chlorate [6,8,[10], [11], [12], [13]], which are both listed as toxic by-products that should be monitored as per the WHO water quality guidelines and have recommended maximum values of 0.7 mg/L in drinking water [14]. Also, ClO_2_ does still result in some formation of halogenated disinfection by-products, but under specific conditions and at lower concentrations than Cl_2_ [6,15]. Finally, there are still some concerns regarding the cost-benefit advantages and the safety and risk management surrounding the implementation of ClO_2_ at water treatment plants [8].

In terms of its aqueous chemistry, ClO_2_ has been shown to be stable over a wide range of pH values (pH 2–10). At pH values below 2, ClO_2_ will undergo two reactions to form chlorate and chlorine (Equations Equation 1, Equation 2)), and at pH values above 9 (Equation (3)) it will form chlorate and chlorite [[16], [17], [18], [19]].

Equation 1 $${4\text{ClO}}_{2}+{4H}^{+}+{4e}^{-}\to {4\text{HClO}}_{2}$$

Equation 2 $${4\text{HClO}}_{2}\;\to {2\text{ClO}}_{2}+{\text{ClO}}_{3}^{-}+{\text{Cl}}^{-}+{2H}^{+}+H_{2}O$$

Equation 3 $${2\text{ClO}}_{2}+{2\text{OH}}^{-}⇌\;{\text{ClO}}_{3}^{-}+{\text{ClO}}_{2}^{-}+H_{2}O$$

At pH values typically used for water treatment processes (e.g. pH 4–8), ClO_2_ acts as an oxidising agent, and can react favourably with various electron donating compounds [[16], [17], [18], [19]]. Overall, the formation of chlorite (Equation (4)) can occur when reducing ClO_2_, and depending on the redox state of the water, chloride formation (Equation (5)) can also occur [16,17].

Equation 4 $${\text{ClO}}_{2}+e^{-}⇌\;{\text{ClO}}_{2}^{-}\;E_{\text{red}}^{o}=0.95\;V$$

Equation 5 $${\text{ClO}}_{2}^{-}+{4H}^{+}+{4e}^{-}⇌{{\text{Cl}}^{-}+2H}_{2}O\;E_{\text{red}}^{o}=1.58\;V$$

The equations, 4 and 5, highlight that the production of chlorite and chloride during the water treatment process with ClO_2_ simply needs electron donating moieties. Examples include nitrites, iron, manganese and NOM species, which are a few of many various inorganic and organic compounds that have been reviewed in the literature, and result in chlorite and/or chloride formation when using ClO_2_ [[16], [17], [18]]. Besides the redox environment, ClO_2_ can undergo photolytic reactions when exposed to UV, and this has been shown to reduce the problematic production of chlorite and halogenated disinfection by-products and enhance the degradation of micropollutants [13,17,20,21].

Despite the use of ClO_2_ in water treatment processes over many decades, there are still a number of unanswered questions and interesting aspects regarding the mechanism and eventual production of different chlorine species and other products when using ClO_2_. When treating natural water or waste water, there are a large number of inorganic and organic chemicals which can react with ClO_2_ and any resulting chlorate and chlorite produced. At the same time, there are several known and unknown chlorine and non-chlorine based intermediates formed, and some recent reviews have highlighted not only the complexity of the main and side reactions in the process, but also the need for further study to identify the fate and behaviour of the chlorine species involved in the overall process [11,13,16,17,20,21].

Prior work has studied the structure and thermochemical properties of various chlorine oxide species using ab initio techniques [22,23] and density functional theory methodologies [24,25]. The current study was undertaken to ascertain the likely formation of any of 60 different chlorine species during water treatment processes, using computational methods. The opportunity was used to highlight the effective contribution derived from a GEMC computational approach, to assist in effectively determining thermochemical properties. The structural presentation of sixty (60) chlorine oxide species that have been identified for this study are listed in Table 1. A limited number of the species considered here, have been exposed to computational processing in the open literature, most probably owing to the complexity of deriving at their optimum structural geometries, tied to the uncertain compliance of basis sets during *Ab Initio* refinement. Many of the chlorine oxide species have been referenced in the Active Thermodynamic Tables [26], also highlighting the existence of a substantial number of these compounds as radical, neutral, anionic and cationic forms.

**Table 1:** Structural presentations and formulae of the selected chlorine oxide species.

Chemical name and formula	Structure	Chemical name and formula	Structure
			
CℓO (g) [26]		[CℓO]^-^ (g) [26]	
Chlorodioxidanyl		Hypochlorite	
	
			
[CℓO]^+^ (g) [26]		CℓOO (g) [26]	
Oxochloronium		Chlorodioxidenium	
	
	
			
[CℓOO]-(g) [26]		[CℓOO] + (g) [26]	
Chlorodioxidanyl		Peroxy hypochlorite	
	
			
	
CℓOCℓ (g) [21]		[CℓOCℓ]^-^ (g) [26]	
Chloro hypochlorite		mu-Oxodichlorate anion	
	
	
			
	
[CℓOCℓ]^+^ (g) [26]		CℓCℓO (g) [26]	
mu-Oxodichlorine cation		Chlorosyl chloride	
	
	
			
	
[CℓCℓO]^-^		[CℓCℓO]^+^	
	
	
	
			
	
CℓCℓ(O)O (g) [26]		CℓOOO (g) [26]	
Chloryl chloride		1-Chloro ozone	
	
	

Chemical name and formula	Structure	Chemical name and formula	Structure
			
	
[CℓOOO]^-^ (g) [26]		Cℓ(O)O_2_ (g) [26]	
1-Chloro ozone anion		2-chloro ozone	
	
	
			
	
[Cℓ(O)O_2_]^-^ (g) [26]		CℓO_3_ (g) [26]	
2-chloro ozone anion		Perchloryl	
	
	
			
	
[CℓO_3_]^-^ (g) [26]		[CℓO_3_]^+^ (g) [26]	
Chlorate		Perchloryl cation	
	
	
			
	
CℓO_4_ (g) [26]		[CℓO_4_]^-^ (g) [26]	
Perchloryl-oxy		Perchlorate	
	
	
			<
	
CℓOCℓO (g) [26]		CℓOOCℓ (g) [26]	
chlorine chlorite		Chloro-oxy hypochlorite	
	
	
			
	
CℓOCℓO_2_ (g) [26]		CℓOCℓO_3_ (g) [27]	
Dichlorine trioxide		Chloro-oxy chlorane trioxide	
	
	
			
	
CℓO_2_CℓO_2_ (g) [28]		CℓO_2_-O-CℓO_2_ [27]	
		Chloryl chlorate	
	
	

Chemical name and formula	Structure	Chemical name and formula	Structure
			
	
CℓOOCℓO_3_ [27]		CℓO_2_-O-CℓO_3_ [27]	
Dichlorine Pentoxide		Chloryl perchlorate	
	
			
	
CℓO_3_-O-CℓO_3_ (g) [27]		CℓO_2_-O-O-CℓO_2_ (g) [27]	
chlorine heptoxide		Chloryloxy chlorate	
	
			
	
CℓO_3_-O-O-CℓO_3_ (g) [11]		[CℓO_3_CℓO_3_]^−2^ [27]	
Perchloryloxy perchlorate		Oxido-[oxido(dioxo)-lambda7-chloranylidene]-dioxo-lambda7-chlorane	
	
			
	
OCℓO (g) [26]		[OCℓO]^-^ (g) [26]	
Chlorine dioxide		Chlorite	
	
			
	
[OCℓO]^+^ (g) [26]		OCℓOO (g) [26]	
Chloryl ion		Chlorine oxide peroxide	
	
			
	
[OCℓOO]^-^ (g) [26]		OCℓCℓO_2_ (g) [26]	
Peroxy chlorite		chlorine perchlorate	
	
			
	
[CℓOH_2_]^+^ (g) [26]		HOCℓ (g) [21]	
Aquachlorine cation		Hypochlorous acid	
	
	
			
	
[HOCℓ]^-^ (g) [21]		[HOCℓ]^+^ (g) [21]	
Hypochlorous acid anion		Hypochlorous acid cation	
	

Chemical name and formula	Structure	Chemical name and formula	Structure
			
	
HOCℓO (g) [21]		[HOCℓO]^-^(g) [21]	
Chlorous acid		Chlorous acid anion	
	
			
	
[HOCℓO]^+^(g) [21]		HOCℓO_2_ (g) [21]	
Chlorous acid cation		Chloric acid	
	
			
	
HOCℓO_3_ (g) [21]		[HOCℓO_3_]^+^ (g) [21]	
Perchloric acid		Perchloric acid cation	
	
			
	
HOOCℓ (g) [21]		[HOOCℓ]^-^ (g) [21]	
Peroxyhypochlorous acid		Peroxyhypochlorous acid anion	
	
			
	
[HOOCℓ]^+^ (g) [21]		HOOCℓO (g) [21]	
Peroxyhypochlorous acid cation		Peroxychlorous acid	
	
			
	
HOOCℓO_2_ (g) [21]		HOOOCℓ (g) [21]	
Peroxychloric acid		Hypochloroperoxoous acid, hydroxy ester	
	
			
	
HOOOOCℓ (g) [26]		HCℓO (g) [21]	
		Chlorosyl hydride	
	
			
	
HCℓO_2_ (g) [21]		HCℓO_3_ (g) [21]	
Chloryl hydride		Chlorine hydride oxide	
	

## 2 Computational strategy

The study employed a three-way approach for the computational work, where both periodic and single molecular models were studied for these species. The aim being to identify the limiting computational conditions to derive thermochemical properties and acceptable criteria to confirm optimum structural geometries. A third approach employed Grand Canonical Ensemble dynamics, as a counter technique to confirm thermochemical energies.

Periodic models were constructed using the MedeA-3.3.1 software, followed by a VASP (DFT) refinement cycle. Molecular models were prepared with the GaussView-6 interface of the Gaussian-16 software suite. The polymer consistent *pcff+* force field was used for the empirical simulations, and was originally designed to describe organic molecules and polymers [29,30]. Essential parameters for a number of these species were absent, to support *Grand Canonical Ensemble* simulations and several force field parameters were added, borrowed and derived from the *cvff* and *pcff* force field repositories. This step established a successful parameterized force field library for all the chlorine species with the exception of HClO, HClO_2_ and HClO_3_ due to inadequate parameters, describing their intra-molecular H-Cl-O interactions. Attempts to utilize the *Reaxff* force field was unsuccessful, due to an overall deficiency in its force field structural parameters for these compounds. No GIBBS (GEMC) simulations were therefore undertaken for these species.

Software programs used for this study included MedeA (GIBBS-9.7.4), MedeA VASP (VASP-6.2.1) [[31], [32], [33]], MedeA (VASP 5.4), MedeA (MOPAC-2016, rev.17.048) [34,35], GaussView-6 [36], Gaussian-16, Rev.C.01 [37], and FactSage-7.3 [38]. Software conditions applied for the individual computational simulations, are presented in Supplementary Sections (S3-S6). The adjusted *pcff+* force field set is presented in Supplementary Tables S2.1–S2.7.

### 2.1 GIBBS ensemble Monte Carlo (GIBBS-9.7.4)

GEMC ensemble dynamics was devised in a periodic environment, incrementally adding single monomeric chlorine oxide species to an adjustable periodic cell, resembling an isobaric-isothermal GIBBS ensemble (GEMC) applying Grand Canonical Monte Carlo (GCMC) simulation [[39], [40], [41], [42], [43], [44]]. It is frequently referred to as an NPT ensemble [45], but in this instance with a stepwise increment of single species to a maximum defined population. The initial cell dimensions were therefore permitted to be adjusted. The Potential Energy (Ui) for each of the models was determined at each step of addition, to monitor the progressive minimum energy, allowing between five to one hundred molecules in a single periodic environment.

The process step followed in the GEMC simulation, was to trap the minimum energy at each incremental addition of (same) species until an inflection in global profile energy was observed. This should then resemble the point in global molecular space, where interactions have been saturated (all inter- and intra-molecular forces compensated for). This could otherwise be described as the stage where multiple inter-molecular ‘spheres’ of interactions, have reached an equilibrium and to some extent, nullify the overall ensemble dipole moment.

The resultant optimum ensemble model were extracted and further subjected to Ab Initio VASP-6.2.1 [31,46] and semi-empirical MOPAC-2016 [34,35] refinement, and some further information is provided in the supplementary section. The potential functions and parameters applied for GEMC simulations are provided in the supplementary section (S1 – S5). A unique set of acceptable Heats of Formation was obtained, correlated against literature reported energies, also stating their electronic and physical structural geometries.

#### 2.1.1 Structure refinements

The structures of all the species in this study were first optimized using VASP hybrid Density Functional Theory (DFT) applying the B3LYP method and a smaller basis set of 6-311G(3df,2p). VASP-6 was applied to both neutral and radical single molecular species within a confined periodic space. Additionally, larger ensemble models were refined to achieve a relaxed periodic unit cell environment. This process guaranteed that the geometry and electronic structure of all species were optimized initially, and the identical starting model structures were utilised across all computational software for comparative analysis.

The optimized structures were then subjected to DFT B3LYP theory, using basis set (cc-pv5z) [47], to determine finite thermochemical properties. Single species models were refined in a constrained periodic cell environment, with the cell dimensions substantially extended, to eliminate any boundary correlations. MOPAC-2016 (Version: 20.302W) considering its full set of Hamiltonians, offered reliable Heats of Formation of the single species and the ensemble derived (optimized) models. For Gaussian-16 [37], an external procedure was applied to derive at thermochemical properties (Supplementary S4.2). Heat Capacities (Cp) and Entropy (S) properties were derived separately.

## 3 Results and discussion

The Heat of Formation of a few species were initially evaluated with Gaussian DFT B3LYP techniques using the 6-311G(3df,2p) and cc-pv5z basis sets. The results indicated that many of the computations failed to converge towards the reference H_f_ values by large margins when employing the 6-311G(3df,2p) basis set. In contrast, a more favourable convergence towards the literature or reference values was observed with the cc-pv5z basis sets (refer to Supplementary Table S11 for comparative data). Therefore, the predominant utilization of the cc-pv5z basis set for heat of formation calculations in this research was justified.

6-311G(3df,2p) is a split-valence Pople basis set that utilizes 6 primitive Gaussian functions to construct the core orbitals that are not involved in chemical bonding. On the other hand, the valence orbitals in the 6-31G(d,p) basis set follow a "triple-zeta" approach where the valence orbitals are triplicated. This strategy of dividing each valence orbital into different "replicas" with distinct primitives, allows for increased flexibility in the size of the final valence orbital upon linear combination, thereby providing the necessary anisotropy in the atomic orbitals to form the molecular orbitals [48].

In contrast, cc-pv5z is a Dunning's correlation consistent basis set (Quintuple-zeta) that exclusively focuses on valence electrons. This basis set eliminates redundant functions and has been optimized for computational efficiency by incorporating progressively larger shells of polarization (correlating) functions compared to 6-311G(3df,2p). As a result, the correlation energy can be more accurately calculated using the cc-pv5z basis set than with the 6-311G(3df,2p) basis set. Additionally, the cc-pv5z basis set contains a greater number of primitive functions than the 6-311G(3df,2p) basis set [49].

Therefore, the cc-pv5z basis set represents a significantly larger basis set that accommodates more functions per atom, enabling more precise handling of large perturbations, core correlations, relativistic effects, and spin-orbit corrections, optimized geometries and calculated enthalpies of formation.

Furthermore, in some cases, the 6-311G(3df,2p) basis set may not converge well due to the hypervalent character of certain chlorine oxide species. To achieve convergence, higher basis sets may be necessary. Kim et al. (1999) [24] observed this while studying the stability of Cl_2_O_3_. They found that some Cl_2_O_3_ isomers have a more hypervalent character than the ClCl(O)O isomer, indicating the need for more extended basis sets, than cc-pvqz for accurate evaluation of relative energies of Cl_2_O_3_.

Gaussian and MOPAC software programs offered optimum molecular geometries, which in turn resulted in reliable Heats of Formation figures for most species. These correlated well with energies extracted from the open literature and are presented in Supplementary Table S13. The thermochemical properties of all chlorine oxide species were determined utilizing both Gaussian and MOPAC programs. Overall, in this study with the 60 selected species, MOPAC performed well and consistently produced relatively more accurate results, than Gaussian. Furthermore, the ease of use and swift computational times of the MOPAC 2016 package, made it a more practical choice when considering functional efficiency when in an environment governed by time constraints.

### 3.1 GEMC analysis

Within the context of the GIBBS Monte Carlo (GEMC) approach, a full descriptive force field library is essential for this technique to portray realistic compound properties. The technique relies on a successive model dynamics simulation, followed by a molecular and total model mechanical minimization, subsequent to each step of incrementally adding a further species molecule. Both the Grand Ensemble model, as well as the individual seeding molecular models were presented in periodic space. A definitive inflection point in ensemble energy (global minima) was observed, at a progressive stage of adding seeding molecular units, different for each ClO_2_ derived species. From this point onwards (adding more seeding molecular units) the ensemble energy remained at a global minimum.

The method resembles an extremely powerful concept, but is reliant on the conditional requirement to provide for a representative force field library, with realistic force field parameters. It can be concluded that the long distance interactions, intrinsic dipole moment compensations, Coulomb contributions, dispersive interactions, besides the fundamental molecular bond and angle components, are invoked. Although the GEMC analysis represents an empirical simulation, it demonstrates the effectiveness of offering optimized models, conditioned to be within the energy realm of Ab Initio techniques. It can therefore be assumed that a stage of ensemble model “saturation” will be reached (more appropriately referred to as a global model equilibrium, encompassing all interactive forces) once the inflection point is reached. This may not represent a meso-scale model status as yet, but proved to be more than sufficient to utilize these scaled (minimized) ensemble models, for further modelling applications.

For the purpose of this study, these ensemble global minimized models were used to derive thermochemical properties, along the same *Ab Initio* conditions applied for single species. Single species and ensemble derived thermochemical properties are reported in Supplementary Table S14. A clear improvement has been observed, for a large number of ensemble model results (applying the GEMC procedure) compared to corresponding single ClO_2_ species entries.

**Fig. 1:**
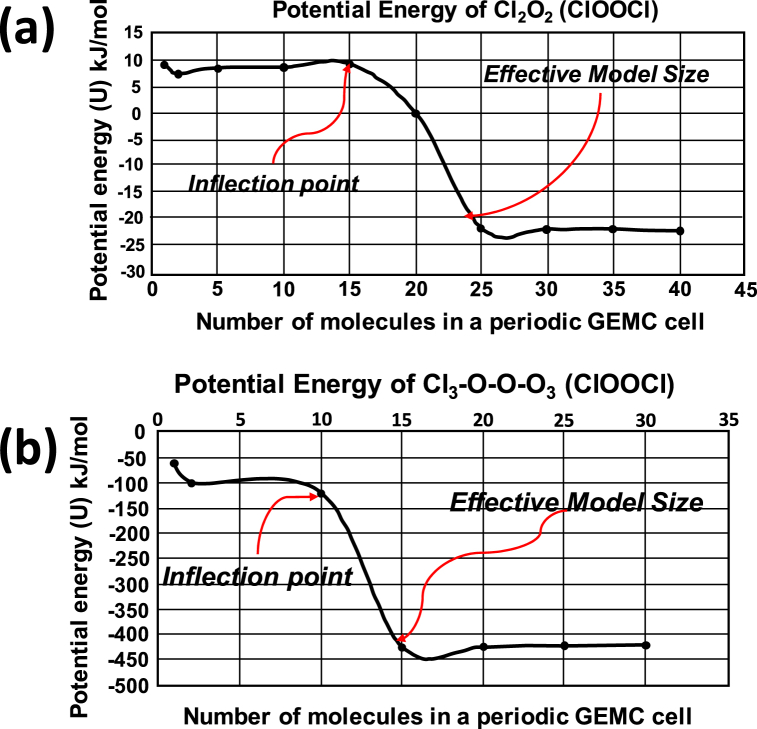
GEMC internal energy profile for Cl_2_O_2_ (a) and ClO_3_-O-O-ClO_3_ (b).

Fig. 1(a) and (b), displays the GIBBS (GEMC) energy profiles for Cl_2_O_2_ and ClO_3_-O-O-ClO_3_ and demonstrate the realistic differences in the inflection point coordinate in these two species. There were 15 species observed for the smaller Cl_2_O_2_ molecule, compared to 10 species noted for the larger ClO_3_-O-O-ClO_3_ species. The GEMC technique furthermore points to the intrinsic differences in the number of molecular units found to reach a global minimum, which in effect represent an effective model size of 25 molecular units for Cl_2_O_2_ compared to 15 molecular units for ClO_3_-O-O-ClO_3_.

These two species are both hydrophilic in nature with negligible dipole moments (Table 3) and direct comparisons can be drawn based on the observed differences above, attributed to; van der Waals, Coulomb (partial charges), Lennard Jones (long distance) and molecular steric contributions, not excluding molecular size. The differences between these two species finally relate to a mere close packing scenario, but with the difference that both molecular geometries are individually optimized, as well as closest molecular interactions. Some of the other species in this series will rely on a different combination of interactions to reach a global minimum. No inter-molecular interactions play a role in single species, which highlights the true value of the GEMC simulations.

**Table 2:** Spin States, Point Groups and Dipole Moments of all ClO2 subspecies. Point Groups and Spin states in parentheses are alternative conditions, derived from Gaussian-16 calculations.

Entry	Molecule formulae	S (J/mol K	Cp (J/mol K)	Point group	Spin State	Dipole moment (Debye)
1	ClO	218.99	21.58	C∗v	Doublet	1.38
2	[ClO]^-^	225.55	25.10	C∗v	Singlet (Triplet)	0.53
3	[ClO] ^+^	224.65	23.72	C∗v	Singlet (Triplet)	1.06
4	ClOO	286.39	40.18	Cs	Doublet (Quartet)	0.64
5	[ClOO]^-^	270.80	35.15	Cs	Singlet (Triplet)	0.89
6	[ClOO] ^+^	283.15	40.11	Cs	Singlet (Triplet)	3.38
7	ClOCl	268.07	39.83	C2v	Singlet	0.59
8	[ClOCl]^-^	281.91	39.12	C2v	Doublet	0.17
9	[ClOCl] ^+^	271.04	38.49	C2v	Doublet	0.76
10	ClClO	273.81	38.73	Cs	Singlet	2.02
11	[ClClO]^-^	274.30	33.30	Cs	Doublet	0.007
12	[ClClO] ^+^	275.27	47.94	Cs	Doublet	2.85
13	ClClO_2_	291.13	50.65	C1	Singlet (Triplet)	1.47
14	ClOOO	307.03	53.99	Cs	Doublet	1.38
15	[ClOOO]^-^	286.06	56.30	C1	Singlet	2.06
16	Cl(O)O_2_	287.55	38.82	C2v	Doublet	0.48
17	[Cl(O)O_2_]^-^	282.91	37.90	C2v	Singlet (Triplet)	5.61
18	ClO_3_	278.92	44.95	C2v	Doublet	0.01
19	[ClO_3_]^-^	277.10	48.40	D3h	Singlet (Triplet)	0.01
20	[ClO_3_] ^+^	280.29	58.17	C2v	Singlet	0.01
21	ClO_4_	293.45	61.02	Td	Doublet	0.00
22	[ClO_4_]^-^	280.73	60.07	C3v	Singlet	1.11
23	ClOClO	293.48	46.50	Cs	Singlet	2.50
24	ClOOCl	305.69	56.79	C2	Singlet	0.00
25	ClOClO_2_	309.92	61.78	C1	Singlet	1.48
26	ClOClO_3_	331.94	78.63	C1	Singlet	2.59
27	ClO_2_ClO_2_	301.36	46.27	D2h	Singlet (Triplet)	0.00
28	ClO_2_-O-ClO_2_	338.35	81.29	C1	Singlet	0.0029
29	ClOOClO_3_	339.98	86.16	C1	Singlet	1.49
30	ClO_2_-O-ClO_3_	355.81	100.76	C1	Singlet	1.36
31	ClO_3_-O-ClO_3_	391.07	118.90	C1	Singlet	0.014
32	ClO_2_-O-O-ClO_2_	338.13	72.75	C1	Singlet	1.11
33	ClO_3_-O-O-ClO_3_	356.58	102.08	C1	Singlet	0.02
34	[ClO_3_ClO_3_]^−2^	319.60	65.55	D3	Singlet (Triplet)	0.00
35	OClO	256.60	33.44	C2v	Singlet (Doublet)	1.90
36	[OClO]^-^	260.29	41.24	C2v	Singlet	2.81
37	[OClO] ^+^	249.60	32.32	C2v	Singlet (Quartet)	0.03
38	OClOO	347.18	57.39	C1	Doublet (Quintet)	0.68
39	[OClOO]^-^	311.88	38.96	C1	Singlet (Triplet)	5.56
40	OClClO_2_	309.84	44.56	Cs	Singlet (Triplet)	2.36
41	[ClOH_2_] ^+^	233.43	29.68	C2v	Singlet (Doublet)	4.02
42	HOCl	242.12	33.48	Cs	Singlet (Quartet)	3.94
43	[HOCl]^-^	258.25	32.17	Cs	Doublet	5.96
44	[HOCl] ^+^	241.45	29.36	Cs	Doublet (Singlet)	1.74
45	HOClO	264.50	39.10	Cs	Singlet (Doublet)	3.73
46	[HOClO]^-^	272.91	33.82	Cs	Singlet (Triplet)	0.01
47	[HOClO] ^+^	269.20	34.96	Cs	Doublet (Singlet)	2.70
48	HOClO_2_	285.88	54.92	Cs	Singlet	0.53
49	HOClO_3_	297.22	65.68	C1	Singlet (Sextet)	3.18
50	[HOClO_3_] ^+^	293.31	39.21	C1	Doublet (Singlet)	2.69
51	HOOCl	263.09	39.76	C1	Singlet (Doublet)	1.73
52	[HOOCl]^-^	299.97	50.45	Cs	Doublet (Quartet)	1.22
53	[HOOCl] ^+^	266.14	36.05	C1	Doublet (Singlet)	2.41
54	HOOClO	293.13	57.38	C1	Singlet	1.10
55	HOOClO_2_	291.48	44.90	C1	Singlet	3.98
56	HOOOCl	278.49	43.02	C1	Singlet	1.64
57	HOOOOCl	320.62	79.13	C1	Singlet (Triplet)	1.68
58	HClO	237.53	29.36	Cs	Singlet	3.94
59	HClO_2_	258.54	36.06	C1	Singlet	2.98
60	HClO_3_	262.97	44.43	C3v	Singlet (Doublet)	2.54

**Table 3:** Selected species extracted to demonstrate their varying partial charge distributions. Mulliken and bonding character in relation to their molecular spin states. Atom connectivity is presented in parenthesis.

Species	Molecular structure	Atomic partial charges	Spin State	Bond Distances (Å) (Atom connectivity)
HOClO		O1: 0.26	singlet	Cl-O1 = 1.51
		Cl: 0.34		Cl-O2 = 1.77
		O2: 0.33		O2-H = 0.98
		H: 0.24		(O1-**Cl**-O2-H)
ClOOCl		Cl1: 0.05	singlet	Cl1**-O1** = 1.82
		O1: 0.03		O1-O2 = **1.32** (Bridge)
		O2: 0.08		Cl2**-O2** = 1.82
		Cl2: 0.06		(**Cl**-O1-O2-**Cl**)
HOOClO		O3: 0.33	singlet	Cl-O3 = 1.51
		Cl: 0.53		Cl-O2 = 1.84
		O2: 0.22		O2-O1 = 1.46
		O1: 0.32		O1-H = 0.98
		H: 0.34		(O3-**Cl**-O2-O1-H)
HOOOCl		Cl: 0.1	singlet	Cl-O3 = 1.81
		O3: 0.09		O3-O2 = **1.35** (Bridge)
		O2: 0.07		O2-O1 = 1.48
		O1: 0.27		O1-H = 0.98
		H: 0.34		(O3-**Cl**-O2-O1-H)
HOOOOCl		Cl: 0.24		Cl-O4 = 1.63
		O4: 0.25		O4-O3 = 1.75
		O3: 0.021	singlet	O3-O2 = **1.26** (Bridge)
		O2: 0.022	(triplet)	O2-O1 = 1.57
		O1: 0.54		O1-H = 0.98
		H: 0.59		(**Cl**-O4-O3-O2-O1-H)
ClOOClO_3_		Cl1: 0.93		Cl1**-O1** = 1.65
		O1: 0.11		**O1-O2** = 1.44
		O2: 0.30		Cl2**-O2** = 1.95
		O3: 0.32	singlet	Cl2-O3 = 1.44
		O4: 0.25		Cl2-O4 = 1.47
		O5: 0.061		Cl2-O5 = 1.44
		Cl2: 0.0056		(**Cl1**-O1-O2-**Cl2**-O3{O4}{O5})
HClO		Cl: 0.41		Cl-O = 1.563
		O: 0.47	singlet	Cl-H = 1.322
		H: 0.064		(H-**Cl**-O)
HClO_2_		Cl: 0.32		Cl-O1 = 1.467
		O1: 0.46		Cl-O2 = 1.467
		O2: 0.46	singlet	Cl-H = 1.361
		H: 0.14		(H-**Cl**-O1{O2})

Species	Molecular structure	Atomic partial charges	Spin State	Bond Distances (Å) (Atom connectivity)
HClO_3_		Cl1: 0.94		Cl-O1 = 1.421
		O1: 0.33		Cl-O2 = 1.421
		O2: 0.33	singlet (doublet)	Cl-O3 = 1.421
		O3: 0.33		Cl-H = 1.327
		H: 0.058		(H-**Cl**-O1{O2} {O3})
ClO_3_-O-ClO_3_		Cl1: 0.99		Cl1-O1 = 1.44
		O1: 0.28		Cl1-O2 = 1.44
		O2: 0.28		Cl1-O3 = 1.48
		O3: 0.28		Cl1-**O4** = 1.90
		O4: 0.26	singlet	Cl2**-O4** = 1.94
		Cl2: 0.99		Cl2-O5 = 1.42
		O5: 0.30		Cl2-O6 = 1.42
		O6: 0.28		Cl2-O7 = 1.46
		O7: 0.28		(**Cl1**-O1{O2){O3}**-O4-Cl2**-O5{O6}{O7})
ClO_2_-O-O-ClO_2_		Cl1: 0.28 O1: 0.20 O2: 0.21 O3: 0.13 Cl2: 0.28 O4: 0.13 O5: 0.21 O6: 0.20	singlet	Cl1-O1 = 1.46 Cl1-O2 = 1.54 Cl1**-O3** = 1.97 O3-O4 = 1.32.(Bridge) Cl2**-O4** = 1.97 Cl2-O5 = 1.46 Cl2-O6 = 1.54 (O1{O2}-**Cl1-O3-O4**-**Cl2**{O5}O6)
ClO_3_-O-O-ClO_3_		Cl1: 1.02 O1: 0.14 O2: 0.30 O3: 0.29 O4: 0.30 Cl2: 1.02 O5: 0.14 O6: 0.29 O7: 0.30 O8: 0.30	singlet	Cl1-O1 = 1.37
				Cl1-O2 = 1.37
				Cl1-O3 = 1.374
				Cl1**-O4** = 1.64
				O4-O5 = 1.36 (Bridge)
				Cl2**-O5** = 1.64
				Cl2-O6 = 1.37
				Cl3-O7 = 1.37
				Cl4-O8 = 1.37
				(**Cl1**-O1{O2){O3}**-O4-O5-Cl2**-O6{O7}{O8})
ClO_2_-O-ClO_3_		Cl1: 0.97		Cl1-O1 = 1.41
		O1: 0.28		Cl1-O2 = 1.43
		O2: 0.25		Cl1**-O3** = 1.82
		Cl2: 0.78		Cl2**-O3** = 1.93
		O3: 0.26	singlet	Cl2-O4 = 1.90
		O4: 0.35		Cl2-O5 = 1.43
		O5: 0.29		Cl2-O6 = 1.77
		O6: 0.31		(**Cl1**-O1{O2}-**O3**-**Cl2**-O4{O5}{O6})

### 3.2 Heats of formation

Table 2 lists the perceived spin-states. Note that doublet and singlet spin states have been observed as the major multiplicities throughout the ClO_2_ series of compounds, resulting from both Gaussian and MOPAC software simulations. It was noticeable that species hosting a higher oxygen content or dual chlorine atoms, displayed definitive spin states of singlet or doublet. Smaller species with lower oxygen content and single chlorine bounded, resulted in variable spin states to be considered. This discrepancy can be attributed to a complex system of bonding order. It should be noted that this variation in spin states, can additionally be resulting from slight variations in electronic structure geometries, portrayed through differences in Molecular Orbital occupancies, during Ab Initio simulation.

Some species are presented as Open Shell structures (see Table 1) resulting in spin polarization, such conditions will result in notable spin multiplicities of doublet, singlet or even higher spin orders. It will no doubt, complicate the selection of appropriate Basis Sets for Ab Initio simulation.

**Fig. 2:**
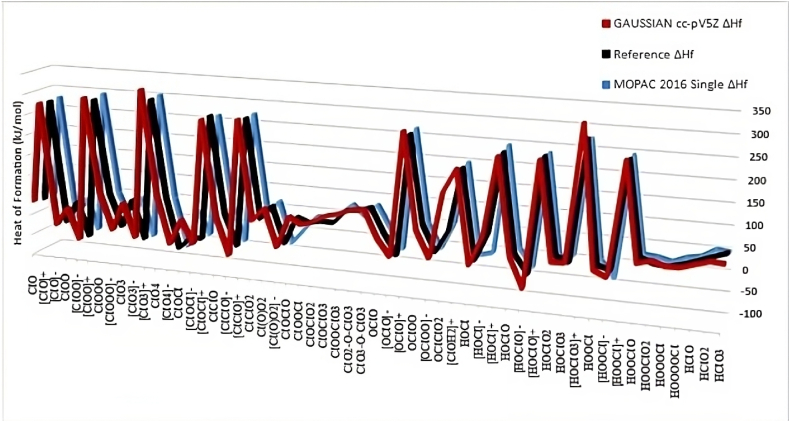
Composite graph of all Heats of Formation energies derived for single molecule species using the indicated computational methods.

The observed variation in spin multiplicities between different species, is further emphasized by their Heats of Formation (Fig. 2) for single ClO_2_ species, applying MOPAC and Gaussian computations. Higher-oxygen hosted species, also containing more than one chlorine atom (exhibiting definitive spin states), have lower Heats of Formation (mid-section of the graph) and are therefore noted to be more stable. This exception also holds for the neutral hydrogen terminated species at the far end of Fig. 2 (adhering to Pauli's exclusion principle) and other sections of the graph displaying higher Heats of Formation, which translates into variable spin multiplicities, for radical and charged species (Hund's rule applies).

Overall, the average of the difference in the heats of formation for all 60 species using Gaussian cc-pv5z and the MOPAC-16 versus the reference values were 8.33 kJ/mol versus −2.81 kJ/mol, indicating that MOPAC-2016 values correlate well with the reference values (Table S13). From the enthalpy values (Table S13), the MOPAC-2016 calculations predict higher stability in general for the 60 species studied. Another general feature from the data (Table S13), was that 13 species had favourable enthalpies of formation using MOPAC-2016, and 16 when using the Gaussian-16 (DFT B3LYP, cc-pv5z). The three compounds with differing trends in the Δ*H*_*f*_ were the molecule [HOCl]^-^ (54.25 vs −9.02 kJ/mol calculated with Gaussian-16 (DFT B3LYP, cc-pv5z) vs MOPAC-2016), compound HOClO_2_ (11.18 vs −3.27 kJ/mol), and the compound HOOCl (2.6 vs −0.68 kJ/mol).The favourable enthalpies of formation suggest that some of the compounds may potentially form during water treatment cycles, and the significance of the structure, geometries and bond lengths to the thermodynamic properties are discussed in the following sections.

### 3.3 Hypervalency of Halogen-O species

Oxygen (O_2_) is considered a bridging bond in the larger ClO_2_ species (refer Table 3) and when considering its electron configuration, there are 2 unpaired electrons in two separate (Пp∗) antibonding molecular orbitals. Bonding with 1 terminal hydrogen atom will neutralize the polarity of one unpaired electron. Chlorine atom exhibits an uneven valence electron configuration, which can similarly attach (share) one remaining O_2_ unpaired electron, resulting in an electron ‘population’ for chlorine, classified as hypervalency, induced by its characteristic strong electronegativity.

Large halogen−O bond distances are characteristic of terminal bonds of halogen and oxygen (imposing a dual influence of electronegativity [this work]) whilst shorter halogen−O bond distances have been found to correspond to hypervalent structures [50].

Lee et al. [51], found that the bonds between multivalent halogen atoms and terminal oxygen atoms undergo tightening of bond distances (shorter bond lengths). This characteristic is observed in all species presented in Table 4.

**Table 4:** Main species products using mole fractions of species-based thermochemistry in aqueous medium applying the FactSage computer software. All species used were the neutral chlorine species from the original 60, and were individually exposed to water.

H_2_O + Reactant	Mol fraction (1:1)		O_2_	Cl [−]	ClOH_2_ [+]	H_2_O
	Water	Reactant				
ClO	1	1	0.32	0.32	0.32	0.031214
ClOO	1	1	0.48	0.24	0.24	0.031222
ClOCl	1	1	0.2	0.4	0.40	0.000013
ClClO	1	1	0.2	0.4	0.40	0.000013
ClClO_2_	1	1	0.33	0.33	0.33	0.000012
ClOOO	1	1	0.58	0.19	0.19	0.031225
Cl(O)O_2_	1	1	0.58	0.19	0.19	0.030783
ClO_3_	1	1	0.58	0.19	0.19	0.031225
ClO_4_	1	1	0.65	0.16	0.16	0.031226
ClOClO	1	1	0.33	0.33	0.33	0.000012
ClOOCl	1	1	0.33	0.33	0.33	0.000012
ClOClO_2_	1	1	0.43	0.29	0.29	0.000012
ClOClO_3_	1	1	0.5	0.25	0.25	0.000012
ClO_2_ClO_2_	1	1	0.5	0.25	0.25	0.000012
ClO_2_-O-ClO_2_	1	1	0.56	0.22	0.22	0.000012
ClOOClO_3_	1	1	0.56	0.22	0.22	0.000012
ClO_2_-O-ClO_3_	1	1	0.6	0.20	0.20	0.000012
ClO_3_-O-ClO_3_	1	1	0.64	0.18	0.18	0.000012
ClO_2_-O-O-ClO_2_	1	1	0.6	0.20	0.20	0.000012
ClO_3_-O-O-ClO_3_	1	1	0.65	0.16	0.16	0.030785
OClO	1	1	0.48	0.24	0.24	0.030781
OClOO	1	1	0.58	0.19	0.19	0.030783
OClClO_2_	1	1	0.43	0.29	0.29	0.000012
HOCl	1	1	0.39	0.39	0.19	0.030761
HOClO	1	1	0.42	0.28	0.28	0.030777
HOClO_2_	1	1	0.54	0.22	0.22	0.030782
HOClO_3_	1	1	0.62	0.18	0.18	0.030784
HOOCl	1	1	0.42	0.28	0.28	0.030777
HOOClO	1	1	0.54	0.22	0.22	0.030782
HOOClO_2_	1	1	0.62	0.18	0.18	0.030784
HOOOCl	1	1	0.54	0.22	0.22	0.030782
HOOOOCl	1	1	0.62	0.18	0.18	0.030784
HClO	1	1	0.19	0.39	0.39	0.030761
HClO_2_	1	1	0.24	0.28	0.28	0.030777
HClO_3_	1	1	0.54	0.22	0.22	0.030782

The molecule ClOOClO_3_, with the atom connectivity entered as Cl-O1-O2-Cl2-O3{O4}{O5} (Table 3) displays two types of O-Cl bonds, a terminal bond between atoms Cl2-O2 at 1.95 Å and a shorter bond of 1.65 Å between the single valence Cl1-O1 bond. The symmetric molecule of ClOOCl has two terminal chlorine bonds, both at 1.82 Å which, as a result of neutralizing (on an equal basis), the unpaired electrons in the unpaired (Пp∗) molecular orbitals in the O-O bond, causes this central O-O bond to be tightened to 1.32 Å.

It must be pointed out that the sharing of different terminal atoms (in the same ClO_2_ species) with the central O-O bond, or in a bonding arrangement with a single oxygen atom, adds to the complexity of having a basis set, capable to distinguish between these bonding orders and to derive at optimum molecular geometries, during *Ab Initio* analyses.

### 3.4 Electronic structure and ClO bonding character

It must be emphasized that chlorine in a hypervalency state, may well migrate its electron dissipation into a limited atomic d-orbital configuration, which further complicates the bonding order and internal Molecular Orbital symmetries involved. This change in stability in the *sp* hybridisation process occurs since the d-shell of chlorine becomes partially occupied which lowers its energy. This trend is clearly visible in the mid-section of Fig. 2, which represents -XO_2_ type molecules such as ClOOCl, ClO2-O-O-ClO2, ClO2-O-ClO3 and the HXO type molecules such as HOOClO, HOOOCl, HOOOOCl, HClO, HClO2 and HClO3. The higher stability of these chlorine oxides is represented by the low Heats of Formation values. Single Cl-O bonds have some ionic characteristics (possibly due to dative bonding), their thermodynamic stability is affected by various factors, one being electronegativity. Examination of the atomic charges of Cl in Cl=O bonds (Table 3) reveals that the poly-oxide species possess highly electropositive charged chlorine atoms. This electro-positivity indicates that these species are prone to chemical attack from radicals and most likely explains why the Cℓ = O bonds are easily dissociated into smaller components (some ionic in nature). Multivalent bonding is usually characteristic of a strong ionic nature [50] that influences thermodynamic stabilisation trends and the structural character of a molecule. Earlier work in the literature proposed that the relative and thermodynamic stability of various halogen molecules resulted from the combination of three factors, these were the electrostatic nature of the halogen-oxygen fragments, the electronegativity of the halogen atom involved, and the degree of halogen valence in the formation of the hypervalent bonds [50].

The factors highlighted in the literature [50], are examined for some of the selected species in this study in Table 3. Table 3 depicts their bond distances and atomic partial charges, demonstrating the intrinsic charge distributions in these complex compounds. The hydrogen terminated species, HOOOOCl, HOOOCl, HOOClO and HOClO all display varying atomic partial charges at the chlorine positions, ranging from 0.96 *ε* to 0.07 *ε*. Larger values for the partial charges are observed for chlorine bound by two oxygen atoms. This large variation can be ascribed to varying bonding character, adopted by the central and neighbouring oxygen atoms. The assignment of two possible spin-states (Singlet and Triplet) for HOOOOCl, can be tied to this vast variation in partial charge distribution. For HOOOOCl, HOOOCl and ClOOCl a bridging double bond is observed (1.26 Å, 1.35 Å and 1.32 Å respectively) between the central two oxygen atoms, clearing the one major paramagnetic contribution, leaving chlorine and/or oxygen atoms on either end, to share a bond in a closed shell condition (as a dative bond). This can result in either a triplet for HOOOOCl or a singlet state, in which case a partial double-bond charge distribution has to be at play with the two atoms neighbouring the central double bond.

It is furthermore significant to observe the large partial charges (0.9689 and 0.8570) for the two chlorine atoms in the two structures (HOClO and HOOClO respectively) flanked on either side by oxygen atoms. The unique assignment of a singlet spin state for both these species, displaying a reduced bond distance of 1.51 Å (a typical Cl-O bond distance) between their terminal oxygen atoms and the chlorine atoms (considered as double bonds) may dictate the residual charge distribution on the inner oxygen atoms. The complex construction of electron sharing and resulting bond character is demonstrated in Table 2, for these species and may be the reason for their vulnerability to dissociate in media such as water, imposing a dipole moment of 1.85 Debye, through dipole-dipole interaction and charge transfer.

### 3.5 Thermodynamic Analysis

A further step in the formal evaluation of ClO_2_ related species' participation in an aqueous medium, was undertaken with the FactSage software [38]. The Gibbs energy minimization module of FactSage, ‘EQUILIB’ which determines the concentration of compounds in an equilibrium state, was used to predict the sustainability of the studied chlorine species in an aqueous medium at standard temperature (T) and pressure (P).

All chlorine species (with confirmed and/or attainable Heats of Formation) were added to the FactSage compound database. This essential process validates the semi-empirical and Ab Initio approach adopted in this study to precisely ascertain the electronic structure, geometries, bonding character and thermochemical properties of the species. The computational outcomes that most accurately converged towards the reference Heat of Formation values were chosen and utilised to input and construct a profile for each species in the FactSage 7.2 program. Selected thermochemical properties Cp, ΔH_f_ and S derived in this study by semi-empirical and Ab Initio supported techniques for single molecules, were substituted into the compound database. Each species was reacted with H_2_O in a mole ratio of water: species of 1:1 and the resultant product species recorded (Table 4). Resultant mole fractions for products, lower than 10^−5^ were discarded.

At the time of this study, the FactSage software (in its current status - Sept. 2022) applying the EQUILIB module, does not support the option to invoke (select) ionic species, but will report on their contribution (prior populating the database via the COMPOUND Module). This imposed a restriction on the analysis of comprehensive ionic chlorine oxide and sub-species analyses, which prompted the alternative approach of exposing individual (neutral) species to water, in a 1:1 mol ratio. A detailed list of a predominant species, resulting from the single species analyses, are listed in Table 4.

The product species that were prevalent in most of the aqueous reactions were O_2_, [ClOH_2_]^+^, Cl^−^, and [ClO_4_]^-^. These species are stable in the aqueous environment and act as precursors for further chlorine oxide interactions. There is limited information from the literature on the predicted product species [ClO_4_]^-^ and [ClOH_2_]^+^, and these maybe previously undetected chlorine species when using ClO_2_. Whereas chloride and oxygen are well reviewed in the literature [[16], [17], [18], [19]].

**Fig. 3:**
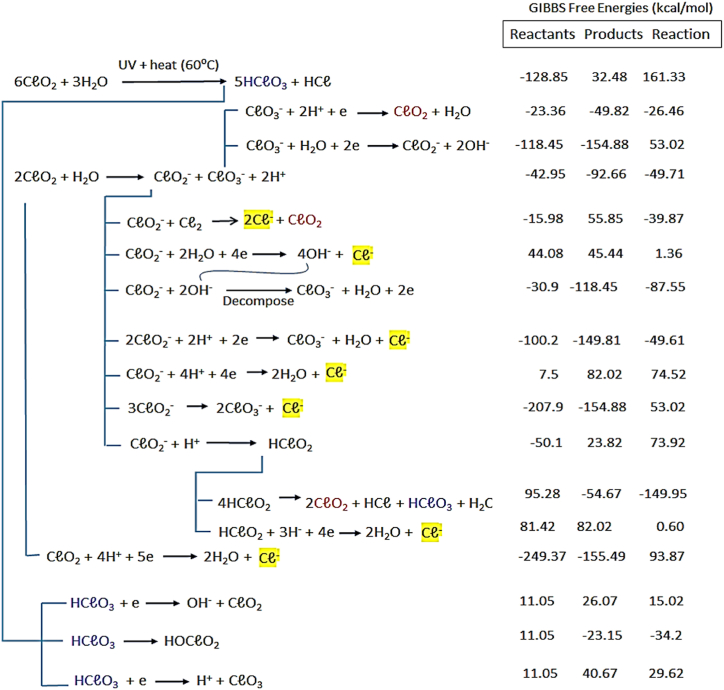
Sequence of the main reactions, some of their inter-dependencies and the distinct formation of Cl^−^, ClO_2_^−^ and ClO_3_ are depicted in an aqueous medium. References applied include [[16], [17], [18],[52], [53], [54]].

From the literature [[16], [17], [18], [19]], the key species formed during the use of ClO_2_ include O_2_, Cl^−^, ClO_2_^−^, and ClO_3_^−^. Using the literature and our results, a plausible set of reaction schemes (Fig. 3) were derived to demonstrate the significant formation of Cl^−^, ClO_2_^−^, and ClO_3_^−^ from selected species used in the current study. The Standard Free Energy of Formation of the individual species (Thermochemical properties in Supplementary Table 4) was used to determine the overall reaction GIBBS Free Energy of Formation of the participating reactions.

It is important to observe the release of chlorine gas, which will require additional precautions to consider, during applications in grand scale processes. The reaction scheme considers the overall ClO_2_ breakdown species in an aqueous medium taking into consideration decomposition and dissociation steps, in both neutral and alkaline aqueous media. The final reaction products of Cl^−^, ClO_2_^−^, ClO_3_^−^ verifies the stable species identified with FactSage with the exception of O_2_. The majority of overall reaction GIBBS Free Energy △G_f_ reveal negative values indicating that reactions are most likely to occur spontaneously.

Overall, from the FactSage analyses we noted that the progressive partial regeneration of ClO_2_ following consecutive reactions, with gradual depletion into other sub-species, is evident as well. Furthermore, the results obtained, identified O_2_, Cl^−^, ClO_2_^−^, and ClO_3_^−^ as the main products in a large proportion of the reactions. Finally, from Table 3 all chlorine oxide species containing more than one chlorine atom (displaying lower Heats of Formation) resulted in the highest product components of O_2_, Cl^−^, ClO_2_^−^, and ClO_3_^−^ as basic precursors for further interaction, also with respect to pathogen control and/or degradation or removal of pollutants in wastewater streams.

## 4 Conclusion

This study demonstrates the importance of extending (GEMC) descriptions of an ensemble model environment, in determining chemical properties and has offered a unique set of acceptable heats of formation. This furthermore validates the electronic structure and geometries of the species investigated.

The work highlights that it is imperative to provide for appropriate force fields to include radical and ionic species in the GEMC procedure. In addition, the derived heat capacity and entropy values can comfortably be applied in further studies, to explore advanced reaction schemes for ClO_2_ and the complex interactions with various residues in aqueous media.

Overall, the basis sets: (6–311++G/(3d2f,3p2d)), (aug-cc-pv5z) and (cc-pv5z) proved adequate to describe the electronic structures of the species during Ab Initio analysis. Also, the MOPAC Hamiltonians, PM6 and PM7 were found to be resilient, in offering confirmed thermochemical properties.

Finally, a few key limitations of the study should be noted. The GEMC simulations were unable to process ionic species, thus the simulations conducted were for neutral species only. Also, the FactSage software (utilised - Sept. 2022) was unable to process ionic species, which may have allowed for the predication of additional unknown species during the reactions with water.

## Data availability

The data used to support the findings of this study are included in the article and within the supplementary information file.

## Funding statement

This study was financially supported by the Faculty of Natural and Agricultural Sciences, at the 10.13039/501100001343University of Pretoria.

## CRediT authorship contribution statement

**Natasha Misheer:** Writing – original draft, Validation, Methodology, Investigation, Formal analysis, Conceptualization. **Patrick G. Ndungu:** Writing – review & editing, Resources, Conceptional. **Jan A. Pretorius:** Writing – review & editing, Validation, Supervision, Software, Resources, Funding acquisition.

## Declaration of competing interest

The authors declare that they have no known competing financial interests or personal relationships that could have appeared to influence the work reported in this paper.
