# Supplementary material for: Semi-empirical supported, Ab initio derived thermodynamic properties for ClO2 and its sub and extended species, applied in water treatment cycles
Source: Heliyon. 2024 Oct 5;10(20):e38796. doi: 10.1016/j.heliyon.2024.e38796 (PMC11620123; doi:10.1016/j.heliyon.2024.e38796)
Supplement: Multimedia component 1 [file mmc1.docx]

**Semi-empirical supported, Ab Initio derived Thermodynamic properties for ClO_2_ and its sub and extended species, applied in water treatment cycles**

Natasha Misheer^1, 2^, Patrick G. Ndungu^1^, and Jan A Pretorius^1,3^

^1^Chemistry Department, University of Pretoria. Private Bag X20, Hatfield, Zip Code 0028, South Africa

^2^ESKOM Research and Innovation Centre, Rosherville, Private Bag 40157, Cleveland,

Johannesburg, South Africa, 2022

^3^Centre for the Advancement of Scholarship, University of Pretoria, Private Bag X20, Hatfield,

Zip Code 0028, South Africa

Corresponding author: Jannie.Pretorius@up.ac.za

**Supplementary Information**

Table S 1: Force Field Atom types defined for the PCFF+ force field parameterisation for chlorine oxide species

| Symbols | Descriptors |
| --- | --- |
| c | - generic sp3 carbon |
| o | - generic sp3 oxygen in alcohol, ether, or acid group |
| h | - generic hydrogen |
| oh | - oxygen bonded to hydrogen |
| ho | - hydrogen bonded to oxygen |
| h* | - hydrogen bonded to fluorine, nitrogen, oxygen |
| cl | – chlorine atom |
| cl4 | – chlorine in ClO_4_^-^ anion |
| o_1r | – oxygen in ClO_4_^-^ anion |
| p | – general phosphorous atom |
| s2 | – sulphur in SO_2_ |
| o1 | – oxygen in NO_2_ |

Table S 2: Equivalence parameters

| Type | NonB | Bond | Angle | Torsion | OOP | Force field reference |
| --- | --- | --- | --- | --- | --- | --- |
| cl4 | cl4 | cl4 | cl4 | cl4 | cl4 | cvff file |
| o_1r | o_1r | o_1r | o_1r | o_1r | o_1r | *cvff* file |
| oh | oh | oh | oh | oh | oh | This work |
| ho | ho | ho | ho | Ho | ho | This work |
| h* | h* | h* | h* | h* | h* | This work |

# S1. Bond Increments parameters

Bond increments describe the charge formed during the bonding of two atoms. The assignment of the atomic charges of a molecule into bond increments, creates a set of general rules, based on the type of atoms in the bond [1]. I and J are the atom types of the bonded atoms. Delta IJ and Delta JI (presented as partial atomic charges measured in elementary charge unit e) refers to the change in atomic charges depending on the direction of the charge i.e., I to J or from J to I. Bond increments parameters are presented in Table S3.

Table S 3: Bond Increment parameters

| I | J | Delta-IJ | Delta-JI | force field  Reference |
| --- | --- | --- | --- | --- |
| cl4 | o_1r | 0.3864 | -0.6364 | pcff file |
| cl | o | 0.3864 | -0.6364 | This work |
| h* | o | 0.4100 | -0.4100 | *pcff* file |
| cl | o_1r | 0.3864 | -0.6364 | This work |

Entries for bond increments: **cl [o]** and **cl [o_1r]** parameters were borrowed from **CVFF** for: **cl4 [o_1r]**. No other parameters have been published to support this study. Bond lengths for **cl [o]** = 1.7 Å, **cl4 [o_1r]** = 1.4 Å and **cl [o_1r]** = 1.6 Å were taken from the reference [2].

# S2. Quartic bond parameters

The quartic bond-stretching potential is presented by:

E = K2 * (R - R0)^2^ + K3 * (R - R0)^3^ + K4 * (R - R0)^4^

where:

- R is the current bond length and R0 the reference bond length in angstroms.
- K2, K3 and K4 are the coefficients for the quadratic, cubic and quartic terms in units of kcal/mol Å^-2^, kcal/mol Å^-3^ and kcal/mol Å^-4^ respectively.

Table S4 presents the Quartic bond parameters used, and in the table I and J are the atom types of the bonded atoms.

Table S 4: Quartic bond parameters

| I | J | R 0 | K2 | K3 | K4 | force field Reference |
| --- | --- | --- | --- | --- | --- | --- |
| cl | o | 1.6500 | 307.0632 | 0.0 | 0.0 | cvff file |
| h | o | 0.9600 | 493.8480 | 0.0 | 0.0 | *cvff* file |
| cl4 | o_1r | 1,4523 | 419.3650 | -838.7299 | 978.5182 | cvff file |
| cl | o_1r | 1.679 | 419.3650 | -838.7299 | 978.5182 | This work |
| cl4 | oh | 1.641 | 419.3650 | -838.7299 | 978.5182 | This work |
| o | o | 1.2080 | 833.6868 | 0,.00 | 0.00 | *cvff* file |
| o | o_1r | 1.3603 | 833.6868 | 0,.00 | 0.00 | This work |
| o_1r | o_1r | 1.3227 | 833.6868 | 0,.00 | 0.00 | This work |

The entries for the **cl [o_1r]** and **cl4 [oh]** parameters were extracted from the **cl4 [o_1r]** entry in PCFF+. The relevant bond lengths (R0) were obtained from literature [2]. The entries for the **o [o_1r]** and **o_1r [o_1r]** parameters were extracted from the **o - o** entry in PCFF+. The relevant bond lengths (R0) were obtained from literature [3].

# S3. Quartic angle parameters

The quartic angle is defined as follows in PCFF*+*:

$\triangle$ = $\boldsymbol{\theta}$ - $\boldsymbol{\theta}$**_0_**

E = K2 * $\triangle$^2^ + K3 *$\triangle$^4^

$\triangle$^3^ + K4 * $\triangle$^4^

where:

- - - $\triangle$ represents Delta which is the change in angle value
    - $\boldsymbol{\theta}$**_0_** is the equilibrium value of the angle.
    - Theta (θ) is the current bond angle, Theta0 (θ _0_) is the reference bond angle in degrees.
    - The usual half factor is included in K [6].
    - K_2_, K*_3_ and* K*_4_* _are_ quadratic force constants in units of: kcal/mol rad^-2^.
    - *i*, *j*, *k* are the atom types involved in the angle.

Table S5 presents the quartic angle parameters used in this study.

Table S 5: Quartic angle parameters

| I | J | K | Theta0 | K2 | K3 | K4 | force field Reference |
| --- | --- | --- | --- | --- | --- | --- | --- |
| o1= | s2= | o1= | 119.300 | 115.2627 | -35.6278 | -26.1261 | *cvff* file |
| o | cl | o | 115.400 | 115.2627 | -35.6278 | -26.1261 | This work |
| * | p | * | 109.5000 | 45.0000 | 0.0 | 0.0 | pcff file |
| o | cl | oh | 109.5000 | 45.0000 | 0.0 | 0.0 | This work |
| * | o | * | 109.5 | 60.000 | 0.0 | 0.0 | *pcff* file |
| cl | o_1r | cl4 | 111,9 | 60.000 | 0.0 | 0.0 | This work |
| cl4 | o_1r | o_1r | 110,3 | 60.000 | 0.0 | 0.0 | This work |
| cl4 | o_1r | o | 110.4700 | 60.000 | 0.0 | 0.0 | This work |
| cl | o | o_1r | 111,74 | 60.000 | 0.0 | 0.0 | This work |
| cl4 | o_1r | cl4 | 119,11 | 60.000 | 0.0 | 0.0 | This work |
| cl4 | oh | ho | 105 | 60.000 | 0.0 | 0.0 | This work |
| o_1r | cl4 | o_1r | 112.2363 | 108.1226 | -24.1496 | -23.3346 | *pcff* file |
| o_1r | cl4 | oh | 104,2 | 108.1226 | -24.1496 | -23.3346 | This work |
| o | cl | o_1r | 104,6 | 108.1226 | -24.1496 | -23.3346 | This work |

Parameters from the SO_2_ (*cvff* file) entry were borrowed for the **o-cl-o** quartic angle. The actual quartic angle from literature [2] was substituted for Theta0. Parameters and Theta0 from the * [p] * entry were borrowed for the **o – cl - oh** quartic angle. The cl was substituted for ‘p’. ‘o’ and ‘oh’ were substituted for *.

Parameters from the * [o] * entry were borrowed for the following quartic angle entries:

- cl [o_1r] cl4 o_1r was substituted for ‘o’. cl and cl4 were substituted for *
- cl4 [o_1r] cl4 o_1r was substituted for ‘o’. cl4 and cl4 were substituted for *
- cl4 [o_1r] o_1r o_1r was substituted for ‘o’. cl4 and o_1r were substituted for *
- cl4 [o_1r] o o_1r was substituted for ‘o’. cl4 and o were substituted for *
- cl [o] o-1r cl and o_1r were substituted for *
- cl [oh] ho oh was substituted for ‘o’. cl was substituted for *
- cl [o_1r] cl4: The quartic angle for cl - o – cl bond was extracted from literature [2] and was substituted for theta0
- cl4 [o_1r] o_1r: The quartic angle for cl - o – o bond was extracted from literature [4] and was substituted for theta0
- cl4 [o_1r] o: The quartic angle for cl - o – o bond was extracted from literature [3] and was substituted for $\boldsymbol{\theta}$**_0_**
- cl [o] o-1r: The quartic angle for cl - o – o bond was extracted from literature [3] and was substituted for $\boldsymbol{\theta}$**_0_**
- cl [oh] ho: The quartic angle for cl -o – h bond was extracted from literature [2] and was substituted for $\boldsymbol{\theta}$**_0_**

Parameters from the o_1r [cl4] o_1r entry were borrowed for the following Quartic angles:

1. o [1r – cl4] oh ! oh was substituted for ‘o_1r’
2. o [cl] o_1r ! o was substituted for ‘o_1r’
3. o_1r [cl4] oh ! Quartic angle was extracted from literature [2] and was substituted for $\theta$_0_
4. o [cl – o] 1r ! Quartic angle was extracted from literature [3] was substituted for $\theta$_0_

# S4. Wilson out of plane parameters

The Wilson out-of-plane potential is defined according to the angle between one bond from the central atom and the plane defined by the other two bonds. The format of the out-of-plane potential (Wilson definition) section is:

E = K * (Chi – Chi0)^2^

where I, J, K and L are the atom types of the four atoms involved in the out-of-plane term, j being the central atom: Chi (χ0) is the reference angle in degrees.

Table S 6: Wilson out of plane parameters

| **I** | **J** | **K** | **L** | **K-Chi** | **Chi0** | force field  **Reference** |
| --- | --- | --- | --- | --- | --- | --- |
| ***** | p | ***** | ***** | 0.000 | 0.000 | *cvff* file |
| o | cl | o | o | 0.000 | 0.000 | *This work* |
| o | cl | o | oh | 0.000 | 0.000 | *This work* |
| o | cl | o | h | 0.000 | 0.000 | *This work* |
| o | cl | o | cl | 0.000 | 0.000 | *This work* |
| cl4 | o_1r | o_1r | cl4 | 0.000 | 0.000 | *This work* |
| o_1r | cl4 | o_1r | cl4 | 0.000 | 0.000 | *This work* |
| cl | o | o_1r | cl4 | 0.000 | 0.000 | *This work* |

Parameters from entry * -p- * - * were used for all Wilson out of plane entries, cl was substituted for p. The relevant atoms were substituted for *.

# S5. Torsion parameters

The three-term cosine expansion of the torsion potential is:

E = ∑(n=1,3) {V(n) * [ 1 - cos(n*Phi - Phi0(n)) ]}

where V1, V2 and V3 are the barrier heights in kcal/mol and Phi (ɸ) is the current torsion angle.

ɸ01, ɸ02 and ɸ03 are the reference torsion angles in degrees. The reference angles are usually 0° or 180°.

The atoms are bonded to each other in the order I-J-K-L.

Table S 7: Torsion parameters

| **I** | **J** | **K** | **L** | **V(1)** | **Phi1(0)** | **V(2)** | **Phi2(0)** | **V(3)** | **Phi3(0)** | force field **Reference** |
| --- | --- | --- | --- | --- | --- | --- | --- | --- | --- | --- |
| * | c | o | * | 0 | 0 | 0 | 0 | 0,13 | 0 | *cvff* file |
| o | cl | o | o | 0 | 0 | 0 | 0 | 0,13 | 0 | *This work* |
| o | cl | o | h | 0 | 0 | 0 | 0 | 0,13 | 0 | *This work* |
| o | cl | oh | ho | 0 | 0 | 0 | 0 | 0,13 | 0 | *This work* |
| o_1r | cl4 | o_1r | cl | 0 | 0 | 0 | 0 | 0,13 | 0 | *This work* |
| o_1r | cl4 | o_1r | cl4 | 0 | 0 | 0 | 0 | 0,13 | 0 | *This work* |
| o_1r | cl4 | o_1r | o | 0 | 0 | 0 | 0 | 0,13 | 0 | *This work* |
| o_1r | cl4 | oh | ho | 0 | 0 | 0 | 0 | 0,13 | 0 | *This work* |
| o | cl | o_1r | cl4 | 0 | 0 | 0 | 0 | 0,13 | 0 | *This work* |
| o | cl | o | oh | 0 | 0 | 0 | 0 | 0,13 | 0 | *This work* |
| o | cl | oh | o | 0 | 0 | 0 | 0 | 0,13 | 0 | *This work* |
| o | cl | o | cl | 0 | 0 | 0 | 0 | 0,13 | 0 | *This work* |
| o | cl | o | o_1r | 0 | 0 | 0 | 0 | 0,13 | 0 | *This work* |
| o_1r | cl4 | o_1r | o_1r | 0 | 0 | 0 | 0 | 0,13 | 0 | *This work* |
| * | o | o | * | 0 | 0 | 0 | 0 | 1 | 0 | *cvff* file |
| cl4 | o_1r | o_1r | cl4 | 0 | 0 | 0 | 0 | 1 | 0 | *This work* |
| cl | o | o_1r | cl4 | 0 | 0 | 0 | 0 | 1 | 0 | *This work* |

# S6. DFT and Ab Initio structure refinement

Computational conditions applied for Gaussian-16 analyses, are presented in Table S8

Table S 8: Parameters applied for Gaussian analysis

| **Gaussian execution conditions** | **Selection** |
| --- | --- |
| Optimization Conditions: | OPT selected plus FREQUENCY |
| Temperature: | 298.15K |
| Pressure: | 1 Atm |
| Spin polarization: Restricted Open Shell HF | ROHF: Charged and Radical Species |
| Spin polarization: Unrestricted Hartree Fock | UHF: Open shell, Radical, Charged species |
| Spin polarization: Restricted Hartree Fock | RHF: Neutral Species |
| Theory: | CCSD |
| Theory: | Hartree Fock |
| basis set: | cc-pv5z |
| Charge: | To specify for charged species |
| Theory: | DFT |
| Functional: | B3LYP, CCSD |
| basis set: | cc-pv5z |
| Spin: | Selected |

The steps used (equations S1 – S5) to extract Energies of Formation, derived from Gaussian-16 applying B3LYP theory were adapted from the reference [5], and the results are presented in Table S 10 – S 11.

The initial step involved calculating the enthalpy of formation (∆_ƒ_H (M, 0 K) for each molecule using the following equations [5]:

| $\Delta_{f}H\left( M,0K \right)=\sum_{atoms} x\Delta_{f}\boldsymbol{H}\left( X,0K \right)- \sum D_{0}\left( M \right)$ | Equation S1 |
| --- | --- |
| $\Delta_{f}H\left( M,0K \right)=\sum_{atoms} x\Delta_{f}\boldsymbol{H}\left( X,0K \right)-\left[ \sum xƐ_{0}\left( X \right)- Ɛ_{0}\left( M \right)- Ɛ_{ZPE}\left( M \right) \right]$ | Equation S2 |
| $=\sum_{atoms} x\Delta_{f}\boldsymbol{H}\left( X,0K \right)-\left[ 627.51\sum xƐ_{0}\left( X \right)- Ɛ_{0}\left( M \right)- Ɛ_{ZPE}\left( M \right) \right]$ |  |

Where the equation parameters are defined as follows:

M = Molecule

X = Elements which make up M

χ = Number of atoms of X in M

∑ D_0_(M) = Atomization energy of the molecule

Ɛ_ZPE_(M) = Zero Point energy of the species

Ɛ_0_(M) = Total energy of the species

H_corr = Thermal correction to Enthalpy

E_tot = Total electronic energy

G_corr = Correction to the Gibbs Free Energy

Multiply by 627.51 = Conversion (Hartree to kcal)

To calculate the enthalpy of formation at 298 K (∆_ƒ_H (M, 298 K) for each molecule, the following equation from the reference [5] were used:

| $\Delta_{f}H\left( M,298K \right)=\Delta_{f}H\left( M,0K \right)+\left[ H_{M}^{0}\left( 298K \right)-H_{M}^{0}\left( 0K \right) \right]-\sum_{atoms} x\left[ H_{x}^{0}\left( 298K \right)-H_{x}^{0}\left( 0K \right) \right]$ | Equation S3 |
| --- | --- |

The Gibbs free energy of formations was determined using the following [5]:

| $\Delta_{f}G\left( M,298K \right)=\Delta_{f}H\left( 298K \right)-298.15\left[ \sum S_{\left( X, 298K \right)}-S_{\left( M, 298K \right)} \right]$ | Equation S4 |
| --- | --- |
| $=\Delta_{f}H\left( 298K \right)-298.15\left[ \sum S_{\left( X, 298K \right)}-627.51(H\_corrM - G\_corrM)/298.15 \right]$ | Equation S5 |

The elemental entropy values used with equation S4 are provided in Table S9.

Table S 9: Elemental Entropu values used with equation S4.

| **Element** | **S_298K_ (Kcal/mol ∙ K)** |
| --- | --- |
| Cl | 39.481 |
| H | 27.418 |
| O | 38.494 |
| C | 37.787 |

Table S 10: Gaussian Electronic energies from a B3LYP calculation

| **B3LYP** | **OClO** | **O** | **Cl** | **H** | **Units** |
| --- | --- | --- | --- | --- | --- |
| Ɛ_0_ | -610.583 | -75.1005 | -460.181573 | -0.50243 | hartree |
| Ɛ_ZPE_ | 0.005783 | 0.00 | 0.00 | 0.00 | hartree |
| E_tot | 0.00894 | 0.0014 | 0.0014 | 0.0014 | hartree |
| H_corr | 0.009884 | 0.0024 | 0.00236 | 0.00236 | hartree |
| G_corr | -0.01926 | -0.0135 | -0.013508 | -0.01065 | hartree |

Table S 11: Gaussian DFT/B3LYP/cc-pv5z/6311G output energies (Hartree) and associated Heats of Formation (kcal/mol)

| **Species** | **Ɛ_0_** | **Ɛ_ZPE_** | **E_tot** | **H_corr** | **G_corr** | **∆H**  **(OK)** | **∆H**  **(298K)** | **∆G (298K)** |
| --- | --- | --- | --- | --- | --- | --- | --- | --- |
| O # (Singlet) | -75.1005 | 0 | 0.001416 | 0.00236 | -0.01495 | 58.99 | 60.03 |  |
| Cl # (Doubet) | -460.182 | 0 | 0.001416 | 0.00236 | -0.01568 | 28.59 | 52.64 |  |
| H # (Doublet) | -0.50243 | 0 | 0.001416 | 0.00236 | -0.01065 | 51.63 | 29.69 |  |
| C # (Singlet) | -37.1984 | 0 | 0.001416 | 0.00236 | -0.01455 | 169.98 | 170.23 |  |
| O ## | -74.9899 | 0 | 0.001416 | 0.00236 | -0.01392 |  |  |  |
| Cl ## | -460.168 | 0 | 0.001416 | 0.00236 | -0.01568 |  |  |  |
| H ## | -0.50226 | 0 | 0.001416 | 0.00236 | -0.01065 |  |  |  |
| C ## | -37.7925 | 0 | 0.001416 | 0.00236 | -0.01351 |  |  |  |
| ClO | -535.377 | 0.002787 | 0.005163 | 0.006107 | -0.01876 | 29.772 | 29.72 | 22.07 |
| [ClO]^-^ | -535.461 | 0.001987 | 0.004407 | 0.005351 | -0.01909 | -23.431 | -23.46 | -31.37 |
| [ClO]^+^ | -534.98 | 0.001753 | 0.004201 | 0.005145 | -0.02037 | 278.19 | 278.18 | 270.94 |
| ClOO | -610.586 | 0.004618 | 0.008553 | 0.009497 | -0.02252 | 21.62 | 21.50 | 6.87 |
| [ClOO]^-^ | -610.707 | 0.004176 | 0.007997 | 0.008941 | -0.02321 | -54.57 | -54.76 | -69.30 |
| [ClOO]^+^ | -610.142 | 0.003875 | 0.00745 | 0.008394 | -0.0227 | 300.13 | 299.79 | 284.57 |
| ClOCl | -995.618 | 0.003612 | 0.007145 | 0.00809 | -0.02235 | 21.46 | 21.03 | 5.11 |
| [ClOCl]^-^ | -995.706 | 0.001233 | 0.005028 | 0.005973 | -0.02604 | -34.99 | -35.26 | -50.19 |
| [ClOCl]^+^ | -995.223 | 0.003982 | 0.007396 | 0.00834 | -0.02244 | 269.53 | 269.03 | 253.32 |
| Cl_2_O | -995.597 | 0.00385 | 0.007412 | 0.008357 | -0.02282 | 35.05 | 34.64 | 19.19 |
| [Cl_2_O]^-^ ***** | -995.645 | 0.002096 | 0.005615 | 0.006559 | -0.02461 | -53.42 | -54.10 | -69.56 |
| [Cl_2_O]^+^ | -995.212 | 0.002828 | 0.006574 | 0.007518 | -0.0246 | 276.12 | 275.83 | 260.96 |
| ClClO_2_ | -1070.79 | 0.009595 | 0.013771 | 0.014715 | -0.01835 | 37.86 | 36.80 | 11.044 |
| ClOOO | -685.709 | 0.007877 | 0.012692 | 0.013636 | -0.02123 | 68.41 | 67.80 | 43.48 |
| [ClOOO]^-^ | -685.851 | 0.007116 | 0.0108 | 0.011744 | -0.02128 | -20.62 | -21.95 | -47.90 |
| Cl(O)O_2_ | -685.707 | 0.00776 | 0.01308 | 0.014024 | -0.02311 | 69.75 | 69.46 | 46.56 |
| [Cl(O)O_2_]^-^ | -685.851 | 0.007469 | 0.011147 | 0.012091 | -0.02004 | -20.63 | -21.95 | -47.99 |
| ClO_3_ | -685.743 | 0.010472 | 0.014074 | 0.015018 | -0.01666 | 49.16 | 47.80 | 21.47 |
| [ClO_3_]^-^ | -685.875 | 0.009585 | 0.013418 | 0.014362 | -0.01711 | -34.08 | -35.31 | -61.76 |
| [ClO_3_]^+^ | -685.292 | 0.00839 | 0.012558 | 0.013502 | -0.01801 | 330.74 | 329.73 | 303.30 |
| ClO_4_* | -760.893 | 0.01041 | 0.014433 | 0.015377 | -0.01675 | 76.79 | 74.64 | 37.12 |
| [ClO_4_]^-^ | -761.083 | 0.014186 | 0.018493 | 0.019438 | -0.01244 | -40.15 | -42.11 | -79.79 |
| ClOClO | -1070.76 | 0.00723 | 0.01139 | 0.012334 | -0.02099 | 56.06 | 54.99 | 29.40 |
| ClOOCl | -1070.79 | 0.006504 | 0.011388 | 0.012332 | -0.02238 | 39.29 | 38.67 | 13.96 |
| ClOClO_2_ | -1145.97 | 0.010652 | 0.015435 | 0.01638 | -0.01879 | 50.56 | 48.83 | 12.93 |
| ClOClO_3_ | -1221.14 | 0.016573 | 0.022351 | 0.023295 | -0.0144 | 67.92 | 65.77 | 19.98 |
| ClO_2_ClO_2_ | -1221.13 | 0.0143 | 0.018233 | 0.019177 | -0.01505 | 73.08 | 69.78 | 21.80 |
| ClO_2-_O-ClO_2_ | -1296.31 | 0.018927 | 0.024792 | 0.025736 | -0.01268 | 84.34 | 81.21 | 24.39 |
| ClO_2_-O-ClO_3_ | -1371.51 | 0.027295 | 0.032621 | 0.033565 | -0.00339 | 89.75 | 85.25 | 16.03 |

| Table-**S11** continued | | |  |  |  |  |  |  |  |  |  |  |  |  |  |  |  |
| --- | --- | --- | --- | --- | --- | --- | --- | --- | --- | --- | --- | --- | --- | --- | --- | --- | --- |
| **Species** | **Ɛ_0_** | | **Ɛ_ZPE_** | | | **E_tot** | | **H_corr** | | **G_corr** | | **∆H**  **(OK)** | | **∆H**  **(298K)** | **∆G (298K)** | |  |
| ClOOClO_3_ | | -1296.33 | | 0.01996 | 0.026008 | | 0.026952 | | -0.01163 | | 75.18 | | 72.17 | | | 15.45 | |
| ClO_3-_O-ClO_3_ | -1446.7 | | 0.030374 | | | 0.038703 | | 0.039647 | | -0.00473 | | 93.30 | | 89.64 | 13.61 | |  |
| ClO_2-_O-O-ClO_2_ | -1371.47 | | 0.023365 | | | 0.031038 | | 0.031982 | | -0.00977 | | 107.20 | | 104.17 | 37.96 | |  |
| ClO_3-_O-O-ClO_3_ | -1521.85 | | 0.037317 | | | 0.044028 | | 0.044972 | | 0.004479 | | 124.17 | | 118.45 | 28.50 | |  |
| [ClO_3_-ClO_3_]^-2^ | -1371.47 | | 0.028141 | | | 0.033538 | | 0.034483 | | -0.00186 | | 110.20 | | 105.74 | 36.14 | |  |
| OClO | -610.583 | | 0.005783 | | | 0.00894 | | 0.009884 | | -0.01926 | | 24.50 | | 23.89 | 7.45 | |  |
| [OClO]^-^ | -610.645 | | 0.003267 | | | 0.006849 | | 0.007793 | | -0.02175 | | -15.96 | | -16.30 | -32.49 | |  |
| [OClO]^+^ | -610.178 | | 0.006108 | | | 0.009185 | | 0.010129 | | -0.01822 | | 278.90 | | 278.24 | 261.30 | |  |
| OClOO | -685.733 | | 0.004705 | | | 0.010421 | | 0.011365 | | -0.02806 | | 51.673 | | 51.63 | 30.17 | |  |
| [OClOO]^-^ | -685.829 | | 0.00475 | | | 0.008655 | | 0.009599 | | -0.02582 | | -8.70 | | -9.88 | -33.85 | |  |
| OClClO_2_ | -1145.81 | | 0.005881 | | | 0.012201 | | 0.013145 | | -0.02631 | | 147.45 | | 146.67 | 113.48 | |  |
| [ClOH_2_]^+^ | -536.291 | | 0.025091 | | | 0.028046 | | 0.028991 | | 0.002482 | | 204.10 | | 202.38 | 179.42 | |  |
| HOCl | -536.045 | | 0.013162 | | | 0.016105 | | 0.017049 | | -0.00978 | | -15.73 | | -16.44 | -31.03 | |  |
| [HOCl]^-^ | -535.926 | | 0.006934 | | | 0.010224 | | 0.011168 | | -0.01455 | | 54.74 | | 54.25 | 38.96 | |  |
| [HOCl]^+^ | -535.637 | | 0.012962 | | | 0.01592 | | 0.016864 | | -0.01055 | | 239.76 | | 239.06 | 224.84 | |  |
| HOClO | -611.193 | | 0.015261 | | | 0.018722 | | 0.019666 | | -0.01037 | | 14.44 | | 13.01 | -11.04 | |  |
| [HOClO]^-^ ***** | -611.066 | | 0.011127 | | | 0.014348 | | 0.015292 | | -0.01570 | | -55.76 | | -57.33 | -80.78 | |  |
| [HOClO]^+^ | -610.834 | | 0.015946 | | | 0.019318 | | 0.020262 | | -0.01012 | | 240.42 | | 238.94 | 215.10 | |  |
| HOClO_2_ | -686.393 | | 0.019286 | | | 0.023362 | | 0.024307 | | -0.00765 | | 13.26 | | 11.18 | -23.15 | |  |
| HOClO_3_ | -761.593 | | 0.027229 | | | 0.031957 | | 0.032902 | | -0.00083 | | 14.84 | | 12.13 | -32.56 | |  |
| [HOClO_3_]^+^ | -761.084 | | 0.016512 | | | 0.022501 | | 0.023446 | | -0.01494 | | 327.65 | | 325.73 | 283.97 | |  |
| HOOCl* | -611.212 | | 0.017467 | | | 0.020866 | | 0.02181 | | -0.00805 | | 4.06 | | 2.60 | -21.57 | |  |
| [HOOCl]^-^ | -611.228 | | 0.011698 | | | 0.016318 | | 0.017262 | | -0.0168 | | -9.90 | | -10.60 | -32.12 | |  |
| [HOOCl]^+^ | -610.812 | | 0.016787 | | | 0.020038 | | 0.020982 | | -0.00924 | | 254.85 | | 253.29 | 229.36 | |  |
| HOOClO | -686.369 | | 0.020306 | | | 0.024864 | | 0.025809 | | -0.00746 | | 29.56 | | 27.78 | -5.719 | |  |
| HOOClO_2_ | -761.551 | | 0.024822 | | | 0.029341 | | 0.030285 | | -0.00357 | | 40.10 | | 37.26 | -7.35 | |  |
| HOOOCl | -686.37 | | 0.021388 | | | 0.025538 | | 0.026482 | | -0.00608 | | 29.47 | | 27.44 | -6.5 | |  |
| HOOOOCl | -761.564 | | 0.022424 | | | 0.028236 | | 0.02918 | | -0.00723 | | 30.21 | | 28.18 | -14.83 | |  |
| HClO | -535.961 | | 0.009811 | | | 0.012762 | | 0.013706 | | -0.01325 | | 34.60 | | 33.90 | 19.39 | |  |
| HClO_2_ | -611.137 | | 0.015657 | | | 0.018874 | | 0.019818 | | -0.00952 | | 49.88 | | 48.31 | 23.82 | |  |
| HClO_3_ | -686.341 | | 0.022859 | | | 0.026355 | | 0.027299 | | -0.00356 | | 48.51 | | 46.07 | 11.05 | |  |

# basis set: B3LYP (cc-pv5z)

## basis set: B3LYP (6-311++G/(3d2f,3p2d))

Basis sets applied:

DFT/B3LYP (cc-pv5z) (Predominantly applied)

DFT/B3LYP (6-311++G/(3d2f,3p2d) *

DFT/B3LYP (aug-cc-pv5z) **

# S7 MOPAC-2016 – (Semi-empirical Ab Initio structure refinement and Heats of Formation)

In this study, the Heats of Formation of the selected single species and ensembles of the various chlorine species was computed using MOPAC-2016 (Version: 20.302W). Computational steps are presented in Table S 12.

Table S 12: MOPAC conditions applied for analysis

| ***MOPAC* execution conditions** | |
| --- | --- |
| Hamiltonians applied: | AM1/MNDO/MNDOD |
|  | PM3/PM6/RM1/PM7 |
| SCF convergence: | 0.01 kcal/mol |
| Structure Optimize: | Normal |
| Wave function: | Automatic |
| Periodic Cell: | Optimize |
| Starting Hessian: | Automatic |
| Convergence: | Normal |
| Iterations: | 10 000 |
| Initial Temp: | 50K |
| Final Temp: | 300K |

Energies of Formation (∆H_ƒ_) of single species selected for this study derived with Gaussian, MOPAC and VASP are presented in Table S 13.

Table S 13: Energies of Formation (∆H_ƒ_) of single species derived with Gaussian, MOPAC and VASP are listed. Models exposed to VASP calculations were confined to periodic cell environments with a Space Group symmetry constraint of P1(1). (Energies displaying severe deviation from Reference entries appear in bold). Thermodynamic S and Cp entries have been derived from the MOPAC ensemble energies

|  | **Literature Reference** | ***Gaussian-16* (DFT B3LYP, cc-pV5Z)** | **MOPAC-2016 (derived)** | **MOPAC-2016 (derived)** | **MOPAC-2016 (derived)** |
| --- | --- | --- | --- | --- | --- |
| **Species** | ***∆H_f_* (298K)** | ***∆H_f_* (298K)** | ***∆H_f_* (298K)** | **S (kJ/mol)** | **Cp (kJ/mol)** |
| ClO | 24.31 [6] | 29.71 | 25.47 | 215.09 | 30.78 |
| [ClO]^-^ | -28.26 [6] | -23.46 | -24 | 215.64 | 31.01 |
| [ClO]^+^ | 274.84 [6] | 278.18 | 276.94 | 213.25 | 21.66 |
| ClOO | 24.56 [6] | 21.5 | 23.28 | 256.64 | 40.54 |
| [ClOO]^-^ | -59.28 [6] | -54.75 # | -53.36 | 265.18 | 46.01 |
| [ClOO]^+^ | 286.71 [6] | 299.79 | 293.61 | 258.75 | 35.69 |
| ClOCl | 18.62 [6] | 21.03 | 19.45 | 264.16 | 43.33 |
| [ClOCl]^-^ | -35.28 [6] | -35.26 | -36.72 | 279.21 | 48.37 |
| [ClOCl]^+^ | 269.14 [6] | 269.03 | 261.74 | 262.36 | 42.76 |
| ClClO | 31.81 [6] | 34.64 | 37.8 | 272.67 | 45.19 |
| [ClClO]^-^ | -44.11 [6] | **-**54.10 # | -43.69 | 293.01 | 49.86 |
| [ClClO]^+^ | 270.09 [6] | 275.83 | 271.85 | 275.27 | 47.94 |
| [ClClO_2_] | 29.16 [6] | 36.79 | 28.96 | 296.4 | 61.96 |
| ClOOO | 53.29 [6] | 67.8 | 45.51 | 297.01 | 52.81 |
| [ClOOO]^-^ | -25.00 [6] | -21.93 | -25.06 | 286.06 | 56.29 |
| [Cl(O)O_2_] | 63.37 [6] | 69.46 | 63.47 | 323.26 | 63.22 |
| [Cl(O)O_2_]^-^ | -25.22 [6] | -21.95 | -35.34 | 300.27 | 62.35 |
| ClO_3_ | 44.46 [6] | 47.79 | 39.31 | 280.33 | 62.65 |
| [ClO_3_]^-^ | -50.83 [6]] | **-35.31** | -55.19 | 264.82 | 56.18 |
| [ClO_3_]^+^ | 297.67 [6] | **329.73** | 300.82 | 273.42 | 35.56 |
| [ClO_4_] | 57.60 [6] | **74.64** | 46.14 | 308.9 | 79.68 |
| [ClO_4_]^-^ | -64.98 [6] | **-42.11** | -72.83 | 265.7 | 63.11 |
| ClOClO | 41.80 [6] | 54.98 | 38.19 | 302.4 | 62.52 |
| ClOOCl | 31.37 [7] | 38.67 | 33.69 | 289.38 | 57.09 |
| ClOClO_2_ | 37.80 [8] | 48.86 ** | 38.45 | 308.11 | 69.63 |
| ClOClO_3_ | 37.40 [9] | 65.77 | 49.52 | 335.93 | 86.6 |
| ClO_2_ClO_2_ | N/R | 69.78 | 50.72 | 340.46 | 96.51 |
| ClO_2_-O-ClO_2_ | N/R | 81.21 | 41.27 | 332.84 | 99.84 |
| ClOOClO_3_ | 69 [9] | 72.17 | 73.94 | 359.09 | 102.89 |
| ClO_2_-O-ClO_3_ | 72.4 [9] | 85.24 * | **47.01** | 367.43 | 111.22 |
| ClO_3_-O-ClO_3_ | 86.2 [9] | 89.64 * | 76.7 | 369.09 | 126.94 |
| ClO_2_-O-O-ClO_2_ | N/R | 104.17 ** | 79.42 | 403.86 | 129.85 |
| ClO_3_-O-O-ClO_3_ | 111.40 [3]^$^ | 118.45 | 108.37 | 471.17 | 164.93 |

Table S 13 continued…

|  | Literature Reference | *Gaussian-16* (DFT B3LYP, cc-pV5Z) | MOPAC-2016 (derived) | MOPAC-2016 (derived) | MOPAC-2016 (derived) |
| --- | --- | --- | --- | --- | --- |
| Species | *∆H_f_* (298K) | *∆H_f_* (298K) | *∆H_f_* (298K) | S (kJ/mol) | Cp (kJ/mol) |
| [ClO_3_-ClO_3_]^-2^ | N/R | 105.74 | 106.06 | 403.99 | 126.56 |
| OClO | 24.36 [6] | 23.89 | 25.47 | 250.18 | 38.97 |
| [OClO]^-^ | -25.74 [6] | -16.29 | -23.14 | 253.39 | 42.54 |
| [OClO]^+^ | 262.13 [6] | 278.24 | 266.34 | 272.24 | 32.61 |
| OClOO | 54.31 [6] | 51.63 | 44.72 | 368.09 | 67.23 |
| [OClOO]^-^ | -8.19 [6] | -9.89 | -12.15 | 302.31 | 65.09 |
| OClClO_2_ | 46.2 [26] | **146.67 #** | 50.13 | 340.18 | 77.86 |
| [ClOH_2_]^+^ | 194.66 [6] | 202.38 | 197.1 | 246.18 | 27.56 |
| HOCl | -18.36 [6] | -16.43 * | -17.81 | 236.16 | 36.05 |
| [HOCl]^-^ | 47.38 [6] | 54.25 | **-9.02 **** | 240.37 | 40.12 |
| [HOCl]^+^ | 237.86 [6] | 239.06 | 244.01 | 235.35 | 35.61 |
| HOClO | 4.94 [6] | 13.01 | 6.77 | 263.6 | 47.48 |
| [HOClO]^-^ | -37.31 [6] | -57.33 # | -34.19 | 283.68 | 57.8 |
| [HOClO]^+^ | 236.82 [6] | 238.93 | 233.62 | 271.55 | 52.3 |
| HOClO_2_ | 10.90 [30] | 11.18 ** | -3.27 | 288.18 | 62.57 |
| HOClO_3_ | 0.43 [6] | 12.13 ** | 3.71 | 307.07 | 74.37 |
| [HOClO_3_]^+^ | 282.36 [6] | 325.73 # | 273.23 | 312.33 | 80.74 |
| HOOCl | -0.32 [6] | 2.60 * | -0.68 | 263.45 | 49.09 |
| [HOOCl]^-^ | -8.12 [6] | -10.6 | **-39.22** | 270.3 | 52.15 |
| [HOOCl]^+^ | 244.70 [6] | 253.27 # | 244.23 | 266.73 | 51.32 |
| HOOClO | 27.30 [10] | 27.78 * | 23.08 | 293.57 | 65.99 |
| HOOClO_2_ | 16.55 [6] | **37.26 **** | 19.25 | 322.75 | 82.36 |
| HOOOCl | 14.00 [10] | **27.44 *** | 11.88 | 286.64 | 58.79 |
| HOOOOCl | 22.70 [11] | 28.18 | 24.78 | 329.09 | 84.55 |
| HClO | 33.24 [6] | 33.89 * | 30.45 | 237.13 | 38.579 |
| HClO_2_ | 45.65 [6] | 48.31 * | 49.06 | 253.22 | 47.44 |
| HClO_3_ | 57.1 [10] | 46.07 * | 45.7 | 264.06 | 50.98 |

N/R - not reported

* Restricted: DFT/B3LYP/cc-pv5z

**Restricted open shell. DFT/B3LYP/cc-pv5z

# Unrestricted: DFT/B3LYP/6-311++G(3d2f,3p2d)

$ Indicates that the ∆Hf (298K) of ClO3-O-O-ClO3 is

calculated from Energies published by Beltran et al.,1999 [5].

Ensemble models were processed in periodic space, extracted from the GIBBS GCMC simulations, and the heats of formation calculated using MOPAC are presented in Table S 14, and compared to literature values.

Table S 14: Heats of Formation of ENSEMBLE models (GEMC derived) calculated with MOPAC

| **Ensemble Models** | **∆*H_f_ (*calculated)** | | | **Literature Reference**  **∆*H_f_* (kJ/mol)** |
| --- | --- | --- | --- | --- |
|  | **MODEL**  **Space** | **MOPAC**  **Hamiltonian** | **∆*H_f_***  **kJ/mol)** |  |
|  | Single | PM6 | 106.56 |  |
| ClO | Ensemble | MNDOD | **91.50** | 101.71 [6] |
|  | Single | MNDOD | 97.40 |  |
| ClOO | Ensemble | MNDO | **101.17** | 102.76 [6] |
|  | Single | AM1 | 81.38 |  |
| ClOCl | Ensemble | AM1 | **82.55** | 77.91 [6] |
|  | Single | MNDOD | 119.83 |  |
| ClClO | Ensemble | RM1 | **143.97** | 31.84 [6] |
|  | Single | PM6 | 121.17 |  |
| ClClO_2_ | Ensemble | MNDOD | **93.47** | 122.00 [6] |
|  | Single | MNDO | 190.54 |  |
| ClOOO | Ensemble | MNDOD | **209.20** | 222.97 [6] |
|  | Single | MNDOD | 263.59 |  |
| Cl(O)O_2_ | Ensemble | MNDOD | **276.73** | 265.14 [6] |
|  | Single | PM6 | 164.47 |  |
| ClO_3_ | Ensemble | RM1 | **173.72** | 185.35 [6] |
|  | Single | PM3 | 211.46 |  |
| ClO_4_ | Ensemble | PM7 | **204.68** | 241.00 [6] |
|  | Single | PM6 | 159.79 |  |
| ClOClO | Ensemble | AM1 | **161.00** | 174.90 [6] |
|  | Single | AM1 | 140.96 |  |
| ClOOCl | Ensemble | AM1 | **127.53** | 131.25 [12] |
|  | Single | MNDOD | 160.87 |  |
| ClOClO_2_ | Ensemble | MNDOD | **164.22** | 158.15 [9] |
|  | Single | PM7 | 207.19 |  |
| ClOClO_3_ | Ensemble | PM6 | **163.18** | 156.49 [9] |
|  | Single | MNDOD | 431.62 |  |
| ClO_2_ClO_2_ | Ensemble | AM1 | **278.45** | Not reported |
|  | Single | RM1 | 211.50 |  |
| ClO_2_-O-ClO_2_ | Ensemble | RM1 | **210.96** | Not reported |
|  | Single | PM7 | 289.66 |  |
| ClOOClO_3_ | Ensemble | PM7 | **275.47** | 288.69 [9] |
|  | Single | MNDOD | 196.52 |  |
| ClO_2_-O-ClO_3_ | Ensemble | PM6 | **281.50** | 302.92 [9] |

| Table-**S14** continued | | | | |
| --- | --- | --- | --- | --- |
| **Ensemble Models** | **∆*H_f_ (*calculated)** | | | **Literature Reference**  **∆*H_f_* (kJ/mol)** |
|  | **MODEL**  **Space** | **MOPAC**  **Hamiltonian** | **∆*H_f_***  **(kJ/mol)** |  |
|  | Single | PM6 | 324.93 |  |
| CℓO_3_-O-CℓO_3_ | Ensemble | PM6 | **330.28** | 345.59 [9] |
|  | Single | PM6 | 332.29 |  |
| CℓO_2_-O-O-CℓO_2_ | Ensemble | AM1 | **439.19** | Not reported |
|  | Single | PM6 | 453.42 |  |
| CℓO_3_-O-O-CℓO_3_ | Ensemble | PM6 | **449.57** | 466.09 [3] ^$^ |
|  | Single | MNDOD | 106.57 |  |
| OCℓO | Ensemble | MNDOD | **84.22** | 101.92 [6] |
|  | Single | PM6 | 111.67 |  |
| OCℓOO | Ensemble | MNDOD | **224.81** | 227.19 [6] |
|  | Single | RM1 | 209.74 |  |
| OCℓCℓO_2_ | Ensemble | RM1 | **207.99** | 193.30 [6] |
|  | Single | PM6 | -74.52 |  |
| HOCℓ | Ensemble | PM6 | **-77.66** | -76.81 [6] |
|  | Single | PM7 | 0.92 |  |
| HOCℓO | Ensemble | PM7 | **27.15** | 20.66 [6] |
|  | Single | MNDOD | -13.31 |  |
| HOCℓO_2_ | Ensemble | MNDOD | **-11.80** | -4.6 [6] |
|  | Single | MNDOD | 15.52 |  |
| HOCℓO_3_ | Ensemble | MNDOD | **13.26** | 1.79 [6] |
|  | Single | RM1 | -2.85 |  |
| HOOCℓ | Ensemble | AM1 | **-3.68** | -1.34 [6] |
|  | Single | RM1 | 92.26 |  |
| HOOCℓO | Ensemble | MNDOD | **94.98** | 90.12 [6] |
|  | Single | PM6 | 80.63 |  |
| HOOCℓO_2_ | Ensemble | MNDOD | **80.88** | 69.24 [6] |
|  | Single | MNDOD | 49.71 |  |
| HOOOCℓ | Ensemble | AM1 | **44.69** | 42.30 [10] |
|  | Single | PM6 | 103.68 |  |
| HOOOOCℓ | Ensemble | PM6 | 90.29 | 94.98 [10] |

$ Indicates that the ∆Hf (298K) of CℓO3-O-O-CℓO3 is calculated from Energies published by Beltran et al. [3]

Table S 15: Calculated Gibbs Free Energies of Formation, obtained with Gaussian-16, applying DFT B3LYP/cc-pv5z

| **Species** | **∆H*_f_* (0K)** | **∆H*_f_* (298K)** | **∆G*_f_* (298K)** |  | **Species** | **∆H*_f_* (0K)** | **∆H*_f_* (298K)** | **∆G*_f_* (298K)** |
| --- | --- | --- | --- | --- | --- | --- | --- | --- |
|  | **(kJ/mol)** | **(kJ/mol)** | **(kJ/mol)** |  |  | **(kJ/mol)** | **(kJ/mol)** | **(kJ/mol)** |
| ClO | 124.64 | 124.41 | 92.40 |  | OClO | 102.58 | 100.02 | 31.19 |
| [ClO]^-^ | -98.10 | -98.22 | -131.34 |  | [OClO]^-^ | -66.82 | -68.24 | -135.99 |
| [ClO] ^+^ | 1164.73 | 1164.68 | 1134.37 |  | [OClO] ^+^ | 1167.70 | 1164.93 | 1094.05 |
| ClOO | 90.52 | 90.02 | 28.76 |  | OClOO | 216.33 | 216.16 | 126.32 |
| [ClOO]^-^ | -228.47 | -229.23 | -290.15 |  | [OClOO]^-^ | -36.43 | -41.32 | -141.72 |
| [ClOO] ^+^ | 1256.58 | 1255.16 | 1191.44 |  | OClClO_2_ | 617.34 | 614.08 | 475.08 |
| ClOCl | 89.85 | 88.05 | 21.39 |  | [ClOH_2_] ^+^ | 854.48 | 847.32 | 751.20 |
| [ClOCl]^-^ | -146.50 | -147.63 | -210.14 |  | HOCl | -65.86 | -68.83 | -129.87 |
| [ClOCl] ^+^ | 1128.47 | 1126.37 | 1060.60 |  | [HOCl]^-^ | 229.19 | 227.13 | 163.12 |
| ClClO | 146.75 | 145.03 | 80.30 |  | [HOCl] ^+^ | 1003.83 | 1000.90 | 941.36 |
| [ClClO]^-^ | -223.66 | -226.51 | -291.23 |  | HOClO | 60.46 | 54.47 | -46.22 |
| [ClClO] ^+^ | 1156.06 | 1154.84 | 1092.59 |  | [HOClO]^-^ | -233.46 | -240.03 | -338.21 |
| ClClO_2_ | 158.51 | 154.03 | 46.22 |  | [HOClO] ^+^ | 1006.59 | 1000.35 | 900.58 |
| ClOOO | 286.42 | 283.86 | 182.04 |  | HOClO_2_ | 55.52 | 46.77 | -96.92 |
| [ClOOO]^-^ | -86.33 | -91.86 | -200.92 |  | HOClO_3_ | 62.13 | 50.79 | -136.32 |
| Cl(O)O_2_ | 292.03 | 290.81 | 194.94 |  | [HOClO_3_] ^+^ | 1371.80 | 1363.77 | 1188.93 |
| [Cl(O)O_2_]^-^ | -86.33 | -91.86 | -200.92 |  | HOOCl | 17.00 | 10.89 | -90.31 |
| ClO_3_ | 205.82 | 200.09 | 89.89 |  | [HOOCl]^-^ | -41.45 | -44.38 | -134.48 |
| [ClO_3_]^-^ | -142.69 | -147.84 | -258.58 |  | [HOOCl] ^+^ | 1067.01 | 1060.47 | 960.28 |
| [ClO_3_] ^+^ | 1384.74 | 1380.51 | 1269.86 |  | HOOClO | 123.76 | 116.31 | -23.95 |
| ClO_4_ | 321.46 | 312.50 | 155.41 |  | HOOClO_2_ | 167.89 | 156.00 | -30.77 |
| [ClO_4_]^-^ | -168.10 | -176.31 | -334.06 |  | HOOOCl | 123.38 | 114.89 | -27.21 |
| ClOClO | 234.71 | 230.19 | 123.09 |  | HOOOOCl | 126.48 | 117.98 | -62.09 |
| ClOOCl | 164.50 | 161.90 | 58.45 |  | HClO | 144.86 | 141.89 | 81.18 |
| ClOClO_2_ | 211.68 | 204.44 | 54.14 |  | HClO_2_ | 208.84 | 202.22 | 99.73 |
| ClOClO_3_ | 284.37 | 275.37 | 83.65 |  | HClO_3_ | 203.10 | 192.89 | 46.26 |
| ClO_2_ClO_2_ | 305.97 | 292.15 | 91.27 |  | H_2_O | -208.34 | -211.18 | -271.43 |
| ClO_2_-O-ClO_2_ | 353.11 | 340.01 | 102.12 |  | CO_2_ | -1717.30 | -1716.67 | -1786,8 |
| ClOOClO_3_ | 314.76 | 302.16 | 64.69 |  | C_2_H_6_ | 102.58 | 100.02 | 356.67 |
| ClO_2_-O-ClO_3_ | 375.77 | 356.92 | 67.11 |  | OH^-^(g) |  |  | -138.79 [33] |
| ClO_2_-O-O-ClO_2_ | 448.82 | 436.14 | 158.93 |  | HCl |  |  | 292.66 [43] |
| ClO_3_-O-O-ClO_3_ | 519.87 | 495.93 | 119.32 |  | [HOO]^-^ |  |  | 1547.02 [43] |
| [ClO_3_ClO_3_]^-2^ | 461.39 | 442.71 | 151.31 |  | Cl^−^ |  |  | -549.31 [33] |

References

1. Yesselman, J.D., et al., *MATCH: An atom-typing toolset for molecular mechanics force fields.* Journal of Computational Chemistry, 2012. **33**(2): p. 189-202.

2. Casper, B., et al., *Molecular Structures of Perchloric Acid and Halogen Perchlorates ClOClO3 and FOClO3.* The Journal of Physical Chemistry, 1994. **98**(34): p. 8339-8342.

3. Beltrán, A., et al., *Structure and Bonding of Chlorine Oxides and Peroxides:  ClOx, ClOx- (x = 1−4), and Cl2Ox (x = 1−8).* The Journal of Physical Chemistry A, 1999. **103**(16): p. 3078-3088.

4. Boese, A.D. and J.M.L. Martin, *Anharmonic force fields of perchloric acid, HClO4, and perchloric anhydride, Cl2O7. An extreme case of inner polarization.* Journal of Molecular Structure, 2006. **780-781**: p. 310-316.

5. Ochterski, J.W. *Thermochemistry in Gaussian*. 2000 [cited 2019 8 June 2019]; Available from: https://gaussian.com/thermo/.

6. Bross, B.R.D.H.n.d. *ATcT Thermochemical Values ver. 1.122.*; Available from: https://atct.anl.gov/Thermochemical%20Data/version%201.122/#ref_5.

7. Klobas, J.E. and D.M. Wilmouth, *UV spectroscopic determination of the chlorine monoxide (ClO)&thinsp;∕&thinsp;chlorine peroxide (ClOOCl) thermal equilibrium constant.* Atmos. Chem. Phys., 2019. **19**(9): p. 6205-6215.

8. Burkholder, J.B., et al., *Temperature dependence of the HNO3 UV absorption cross sections.* Journal of Geophysical Research: Atmospheres, 1993. **98**(D12): p. 22937-22948.

9. Sicre, J.E. and C.J. Cobos, *Thermochemistry of the higher chlorine oxides ClOx (x=3, 4) and Cl2Ox (x=3–7)†.* Journal of Molecular Structure: THEOCHEM, 2003. **620**(2): p. 215-226.

10. Francisco, J.S. and S.P. Sander, *Existence of a Chlorine Oxide and Water (ClO.cntdot.H2O) Radical Complex.* Journal of the American Chemical Society, 1995. **117**(39): p. 9917-9918.

11. Clark, J. and J.S. Francisco, *Study of the Stability of Cl2O3 Using ab Initio Methods.* The Journal of Physical Chemistry A, 1997. **101**(38): p. 7145-7153.

12. Thorn, R.P., et al., *Ionization Energy of Cl2O and ClO, Appearance Energy of ClO+ (Cl2O), and Heat of Formation of Cl2O.* The Journal of Physical Chemistry, 1996. **100**(33): p. 14178-14183.
